# Supplementary material for: Largest baleen whale mass mortality during strong El Niño event is likely related to harmful toxic algal bloom
Source: PeerJ. 2017 May 31;5:e3123. doi: 10.7717/peerj.3123 (PMC6055221; doi:10.7717/peerj.3123)
Supplement: Supplemental Information 1 [file peerj-05-3123-s001.pdf]

INFORME TÉCNICO SOBRE LA MORTALIDAD MASIVA DE BALLENAS EN PUERTO SLIGHT Y CALETA BUENA, GOLFO DE PENAS, REGIÓN DE AYSÉN (EXPEDICIÓN DE MAYO 2015)

Rodrigo Hucke-Gaete, Instituto de Ciencias Marinas y Limnológicas, Universidad Austral de Chile (ICML, UACH) / Centro Ballena Azul (CBA).

Francisco Viddi**,** World Wildlife Fund, Chile (WWF Chile).

David Cassis, Universidad Santo Tomas, representante para Sudamérica de Scotia Rapid Testing LTD.

Luis Bedriñana (ICML, UACH; CBA).

Verena Häussermann, Centro Científico Huinay.

M. José Pérez-Alvarez, Instituto de Ecología y Biodiversidad (IEB), Facultad de Ciencias, Universidad de Chile/ Centro de Investigación Eutropia.

Fanny E. Horwitz, Programa de Magíster en Ciencias Biológicas, Facultad de Ciencias, Universidad de Chile.

Carolina S. Gutstein, Red Paleontológica Universidad de Chile, Lab. Ontogenia y Filogenia, Facultad de Ciencias, Universidad de Chile/Paleobiology Dept., NMNH, Smithsonian Institution.

Gabriela Garrido-Toro, Tesista Universidad Austral de Chile, Valdivia; Asociación de Investigadores Museo de Historia Natural Río Seco, Punta Arenas, Chile

Benjamín Cáceres, Pasante en Mamíferos Marinos, Instituto Antártico Chileno, Punta Arenas (INACH); Asociación de Investigadores Museo de Historia Natural Río Seco, Punta Arenas, Chile

Anelio Aguayo, Departamento científico, Instituto Antártico Chileno, Punta Arenas (INACH); Asociación de Investigadores Museo de Historia Natural Río Seco, Punta Arenas, Chile

Mauricio Ulloa, Servicio Nacional de Pesca y Acuicultura, Chile.

1) ANTECEDENTES GENERALES

Casi un 50% de las especies existentes en el mundo habitan o visitan las costas de Chile. Una de las especies emblemáticas que visitan las aguas de la costa chilena incluye la ballena boba o rorcual de Rudolphi (*Balaenoptera borealis*) o sei Whale en Inglés. Esta especie se encuentra distribuida en todos los océanos del mundo (Rice 1998), sin embargo existe poco conocimiento acerca de los límites de sus poblaciones y de sus patrones migratorios.

Así como otras grandes ballenas de barbas, datos obtenidos durante la caza ballenera suponen una migración latitudinal según la estación del año (alimentación durante el verano en aguas sub-antárticas y reproducción durante el invierno en aguas tropicales). Este patrón migratorio en conjunto con otras características ecológicas, biológicas y geográficas resultaría en una separación temporal de los diferentes grupos o subpoblaciones de ballenas de Rudolphi de los dos hemisferios. Sin embargo estudios genéticos no han definido claramente la separación de las poblaciones de los dos hemisferios (Kanda et al. 2006). Para propósitos de manejo las poblaciones han sido separadas en la base de las diferentes características biológicas y de migración como en las otras ballenas de barbas. En el hemisferio sur se asumen seis poblaciones (Donovan 1991), mientras que en el Pacifico norte dos o tres poblaciones y en el Atlántico norte existirían alrededor de ocho poblaciones (solo tres son consideradas para propósitos de manejo).

El tamaño de la población de la ballena Boba o Rorcual de Rudolphi (*Balaenoptera borealis*), "Sei Whale", en el Hemisferio Sur, fue calculado en unos 191.000 ejemplares para la década de los 40', antes de iniciarse sus capturas comerciales. Posteriormente, al cese de las capturas comerciales, en el año 1984, el Comité Científico de la Comisión Ballenera Internacional, calcularon una población de 37.000 ejemplares para nuestro Hemisferio (Gambel 1985).

En aguas marinas Chilenas continentales, entre los años 1929 y 1983, las capturas de esta especie constituyeron el 17.3% del total de los Misticetos capturados, después del rorcual común y del rorcual gigante (Aguayo Lobo 1974). Los avistamientos de ballenas bobas o rorcuales de Rudolphi en aguas adyacentes al Golfo de Penas (44° - 48°S), se conocen desde hace mucho tiempo, así Aguayo-Lobo (op. Cit.), registró en Marzo de 1966, alrededor de 244 ejemplares; en Octubre del mismo año, 399 y en Diciembre del mismo año, 142 animales. Dos años después, los balleneros japoneses que trabajaban en Chile, exploraron las aguas marinas entre los 40° y los 48°S, registrando muchos ejemplares de esta especie e informaron que la mayor concentración de ellos se encontraron en aguas adyacentes a la Península de Taitao, en el límite Norte del Golfo de Penas, donde se registraron cientos de ejemplares y una gran abundancia de plancton (Pastene y Schimada 1999). Dichos autores concluyeron que *Balaenoptera borealis* se concentra en los meses de verano en aguas del Golfo de Penas para alimentarse. Posteriormente, en Mayo del 2001, Aguayo et. al; sugiere que esta especie incursiona los canales exteriores cercano al océano Pacífico por protección o como posibles áreas de alimentación permanente u ocasional durante sus migraciones. Los registros recientes de los investigadores de la Universidad Austral de Chile, informan de un número creciente de avistamientos del rorcual de Rudolphi en las aguas

correspondientes a las regiones de Los Lagos y de Aysén (43°-49°S). En consecuencia, después de 32 años del cese de las capturas comerciales de *Balaenoptera borealis* en Chile, la especie estaría recuperando lentamente el tamaño de su población en aguas del Pacífico Sur Oriental. Sin embargo, la muerte de 39 ejemplares en el lapso de varios meses en el Golfo de Penas, es un fenómeno digno de estudiarse. Afortunadamente, en los cuatro ejemplares estudiados durante la Expedición al Golfo de Penas, en Mayo del presente año, por el grupo de Punta Arenas, no se encontraron signos de interacción humana con los animales muertos en dicha región de concentración histórica de esta especie.

Estudios sistemáticos y recientes llevados a cabo por investigadores de la Universidad Austral de Chile, y el Centro ballena azul, dan cuenta de un creciente número de avistamientos en el área del sur de Chile (región de los Lagos y Aysén). Situación similar ocurre en la región austral de Chile, donde los avistamientos han ido en aumento (Fundación CEQUA).

En mayo de 2015, la Dra. Verena Häussermann, del Centro Científico Huinay, realizó una denuncia sobre la muerte masiva de ballenas en el sector de puerto Slight y golfo de Penas luego de constatar dicha ocurrencia durante el desarrollo de investigaciones sobre invertebrados marinos en los dias 21 y 22 de abril. Producto de lo anterior, la Armada de Chile puso a disposición de la investigación el PSG Ortiz, con el objeto de realizar una expedición a la zona, junto a un grupo multidisciplinario de profesionales y científicos de la Universidad Austral de Chile, Universidad de Chile, Instituto Antártico Chileno, Asociación de Investigadores del Museo de Rio Seco, Fundación CEQUA, Centro Científico Huinay, WWF Chile, Centro Ballena Azul y la Brigada Investigadora de Delitos Contra el Medioambiente (BIDEMA) de la PDI, medios de prensa de TVN y Megavisión, bajo la coordinación de Sernapesca.

En general, los varamientos son reconocidos como eventos importantes de los cuales importante información científica puede ser recolectada. Se ha demostrado la existencia de nuevas especies, provisto de información sobre tasas de crecimiento, reproducción, hábitos alimenticios, estructura poblacional y factores de mortalidad, tales como toxinas, contaminantes e infecciones. Recientemente, información obtenida de animales muertos y varados ha incrementado el entendimiento de los impactos de sonares y detonaciones submarinas. Así mismo, sobre largo periodos de tiempo, los varamientos han demostrado incrementar el conocimiento sobre la diversidad y ensamble de especies

Los mamíferos marinos, como especies depredadoras de nivel trófico medio, son centinelas y especies indicadoras de la salud de los ecosistemas. De esta forma, determinar las causas de muerte y varamiento de mamíferos marinos es de esencial importancia porque nos asiste a entender de mejor manera la biología de los animales en cuestión. Así mismo, en caso de que las muertes hayan sido provocadas directa o indirectamente por acciones y/o actividades humanas, la información recopilada puede ser de gran utilidad para generar y desarrollar planes de acción y contingencia para evitar y mitigar los impactos de dichas actividades.

En el marco de la investigación formal asociada a la mortalidad de ballenas en el Golfo de Penas en la Región de Aysén, el siguiente informe entrega los antecedentes técnicos y científicos junto con las hipótesis de posibles causas de la mortalidad de estos animales en el área de interés.

2) ANTECEDENTES NORMATIVOS

Los recursos o especies hidrobiológicas, entendiendo por tales a toda especie de organismo en cualquier fase de su desarrollo, que tenga en el agua su medio normal o más frecuente de vida, y sus ecosistemas están sometidos a la soberanía del Estado de Chile en las aguas terrestres, aguas interiores y mar territorial, así como a sus derechos de soberanía y jurisdicción en la Zona Económica Exclusiva y en la Plataforma Continental, de acuerdo a las normas de derecho internacional y a las de la Ley General de Pesca y Acuicultura (LGPA).
En conformidad a la soberanía, a los derechos de soberanía y a su jurisdicción, el Estado de Chile tiene el derecho de regular la exploración, explotación, conservación y administración de los recursos hidrobiológicos y sus ecosistemas existentes en todos los espacios marítimos antes mencionados.
De acuerdo con esto, el Estado de Chile podrá autorizar la exploración y explotación de los antes mencionados recursos hidrobiológicos existentes en los espacios referidos, sujeto a las disposiciones de la LGPA.

En el contexto de los varamientos y tomas de muestras biológicas de ejemplares varados muertos, el Artículo 135 bis de la citada Ley establece que no tendrá responsabilidad legal el que tenga , posea o transporte ejemplares muertos, partes de estos o sus derivados siempre que cuente con un permiso otorgado por el Servicio Nacional de Pesca y Acuicultura. Dicha autorización solo podrá ser otorgada a instituciones de educación reconocidas por el Estado, museos y centros de investigación y conservación marina ubicados en el territorio nacional que tengan fines de docencia, investigación, depósito o exhibición.

Finalmente, es necesario dejar claro que conforme lo establecido en el Decreto con Fuerza de Ley N° 5 de 1983, del Ministerio de Economía, Fomento y Reconstrucción, le corresponde al Servicio Nacional de Pesca y Acuicultura ejecutar la política pesquera y acuícola y fiscalizar su cumplimiento.

3) ACCIONES Y BITACORA DE EXPEDICION

a) Pre-crucero

Bajo la coordinación del Servicio Nacional de Pesca, los investigadores seleccionados e invitados a participar en la expedición entregaron un documento indicando sus intereses y la experticia que pondrían a disposición en la investigación formal ejecutada a petición de Fiscalía Región de Aysén. Dos reuniones formales fueron ejecutadas para establecer los objetivos y metas de la expedición, comprender los antecedentes y limitantes, distribución de roles y responsabilidades, y finalmente entender los protocolos formales de toma de datos (fotografías, datos de ubicación GPS, fotografías y muestras de tejido), almacenamiento y posterior distribución.

b) Durante el crucero

La implementación acciones de muestreo biológico siguió un patrón de acciones jerárquicas para desarrollar la evaluación de la manera más eficiente y ordenada posible. Las actividades concretas fueron desarrolladas por equipos de personas especializadas.

La expedición se desarrolló a través de cinco componentes o etapas:

1. Una exploración preliminar rápida junto al equipo de expertos de la BIDEMA mediante una embarcación menor (de tres a cuatro horas) para determinar el escenario de trabajo, numero de ballenas muertas, su estado de descomposición y marcaje (etiqueta visible con número). Un equipo de tres personas junto a la PDI recorrió el área en un zodiac para aproximarse a cada uno de los animales muertos con el objeto de caracterizar preliminarmente el evento, marcando a cada individuo con una bandera/ficha identificadora, determinar especie, obtener su posición geográfica mediante GPS, establecer su orientación y sacar fotografías generales y específicas. Estas fotografías fueron tomadas de todo el animal, cabeza, zona genital, aleta caudal, aleta dorsal, mandíbula y cicatrices, cuando fuese posible. En este primer recorrido, se intentó determinar sexo y longitud total de la mayor cantidad de individuos posibles, estableciendo el estado de descomposición de cada uno.
2. Desarrollo de un muestreo de primer nivel en aquellos animales (cuatro), cuyo acceso fue posible para la obtención de medidas estándar y muestras de encías, piel y músculo desde una sección anterior a la aleta dorsal en todos los casos y preservadas en alcohol al 70%. En esta etapa se extrajeron también muestras de barbas, otros tejidos (de órganos) y parásitos. Así mismo, se extrajeron fluidos gastro-intestinales con el objeto de realizar pruebas cualitativas *in situ* para la detección de biotoxinas (paralizante y amnésica mediante tests rápidos basados en anticuerpos producidos por Scotia Rapid Testing Ltd.) . Estas tareas fueron realizadas por personal experimentado, utilizando medidas de seguridad acorde (*e.g.* doble guante anticorte, mascarilla y gafas de seguridad).
3. Desarrollo de un circuito costero en una embarcación menor (zodiac) para la obtención de tejido (principalmente piel, musculo, hueso y barbas) de todo el resto de ballenas muertas encontradas. El protocolo siguió los estándares mencionados en paso 2).
4. Obtención de muestras de agua y potenciales vectores de biotoxinas asociadas a floraciones algales nocivas (almejas y choritos, Figura 1); y
5. Búsqueda de más cuerpos en playas durante la travesía de regreso. Posición o muestras de tejido no fueron obtenidas para la mayoría de los animales vistos desde el buque de la armada.
6. Análisis de muestras de vectores de biotoxinas y contenido estomacal mediante tests rápidos para detección de VAM y VPM.

Dentro de lo posible, las muestras fueron almacenadas debidamente en alcohol en recipientes de plástico y bolsas, rotuladas ordenadamente. La ficha de datos presenta información de cada

ballena, su posición, su estado de descomposición y una batería de datos que indica el tipo de muestra (y su cantidad) obtenida. Cabe destacar, que dependiendo del estado de los cuerpos, se pudo obtener solo algunos de los tejidos.

Las muestras de agua para determinación de presencia de fitoplancton tóxico mediante microscopía fueron preservadas con solución de Lugol (solución saturada de iodo) para su análisis posterior ex situ en la Universidad Santo Tomás, sede Santiago.

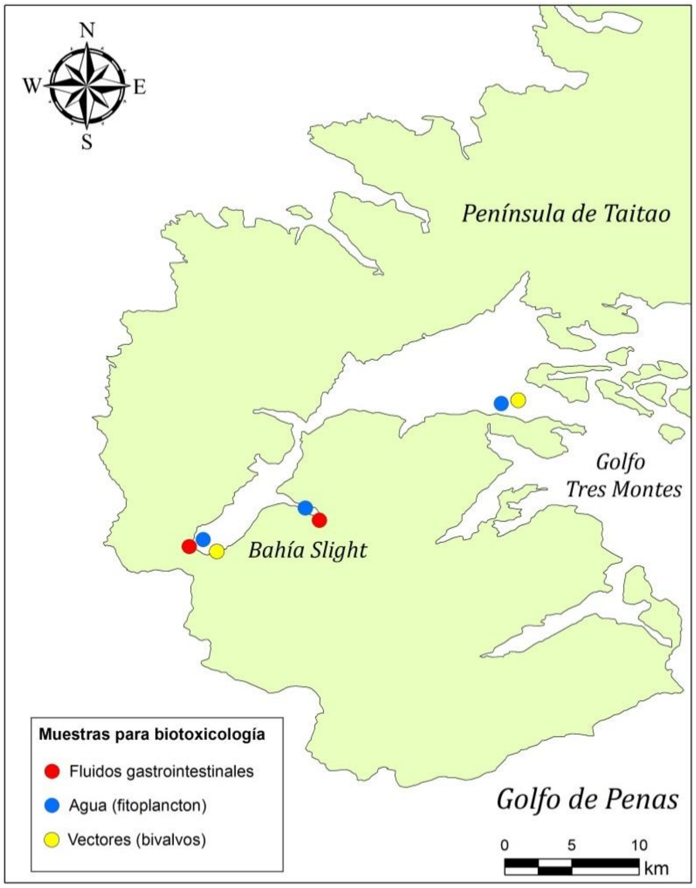

Figura 1. Sitios de toma de muestras de contenidos estomacales e intestinales de ballenas, muestras de agua y de bivalvos vectores de biotixnas de floraciones algales nocivas (choritos y almejas).

c) Actividades post-crucero

Una vez de regreso a puerto, las muestras fueron trasladadas a la Universidad Austral de Chile, Valdivia, para su almacenamiento en un congelador apropiado. Las muestras se encuentran bajo resguardo formal emitido por SERNEPASCA. A la fecha se ha desarrollado una sola reunión para la coordinación de análisis de muestras el día 9 de junio, distribución de las mismas y desarrollo de informe preliminar de la expedición.

Las muestras de agua y contenido gastro-intestinal fueron trasladadas a la Universidad Santo Tomás, sede Santiago, y analizadas mediante microscopía.

Una de las muestras de periótico fue trasladada a la Facultad de Ciencias, Universidad de Chile en Santiago, para la realización de tomografía volumétrica computada de manera de analizar la anatomía interna en búsqueda de posibles daños auditivos (microfractura ósea en el oído interno).

4) DESCRIPCION Y CARACTERIZACIÓN DE LA MORTALIDAD

a) Numero de animales muertos encontrados y muestreados

Para la determinación del número mínimo de individuos (NMI) se utilizó el conteo del número de cráneos (considerando que las carcasas mantenían los cráneos articulados y, en un caso, con un cráneo asociado a esta). Se observaron 39 restos de ballenas pertenecientes al menos a tres eventos de mortalidad distintos (carcasas y restos de esqueletos, Figura 2). Las ballenas muertas recientemente, con una data aproximada de 2-3 meses presentaban un estado de descomposición que exponía tejidos internos como la grasa, musculo, y hasta restos de órganos y contenidos estomacales. Sin embargo, en algunos casos aún se observaban restos de piel en los individuos. En otros casos, solo se encontraron restos óseos, si preservación de tejido blando, denotando un mayor antigüedad de estos restos (ver tabla 2). Se determinó que la presencia de un cráneo (o buena parte de él) representaba un individuo en particular. Un total de 22 ballenas distintas fueron muestreadas (tabla 1, además ver adjuntos con base de datos). El resto de cuerpos no fueron muestreados por estar fuera del alcance (cuerpos vistos desde el buque en traslado de regreso) o porque se trataba de cráneos solamente.

Se registró la posición de orientación de las carcasas en las playas, las cuales fueron ploteadas en un diagrama de rosa (Figura 3), utilizando el programa PAST (3.07)[1]. Se puede observar una dirección preferencial de las carcasas en los cuadrantes NE-SW (14 de 18) aunque esta tendencia no fue significativa en el test post hoc de von Mises (p=0,08), probablemente debido al pequeño numero de muestra y también a la presencia de patrones de corrientes, vientos y mareas complejos dentro del canal.

1 Hammer, Ø., Harper, D.A.T., and P. D. Ryan, 2001. PAST: Paleontological Statistics Software Package for Education and Data Analysis. Palaeontologia Electronica 4(1): 9pp.

Tabla 1. Resumen de número de restos de ballenas encontradas y ballenas muestreadas en bahía Slight y zonas aledañas.

MORTALIDAD DE BALLENAS (ACTUALES Y PREVIOS)	n
Número de ballenas recientes (evento mortalidad aproximado: marzo-abril)	29
Numero de ballenas antiguas solo con presencia de restos óseos (cráneos)	10
Numero de ballenas de los cuales se obtuvo alguna muestra (de todos los eventos)	22
**Número total de ballenas encontradas**	**39**

Tabla 2 – Condición de preservación de las carcasas encontradas en bahía Slight y zonas aledañas.

**Condición de la carcasa**	***n***
Completa e intacta	3
Completa, huesos expuestos	8
Desarticulada, no intacta	2
Completamente desarticulada, asociada	5
Huesos aislados (cráneos, vertebras, etc)	13

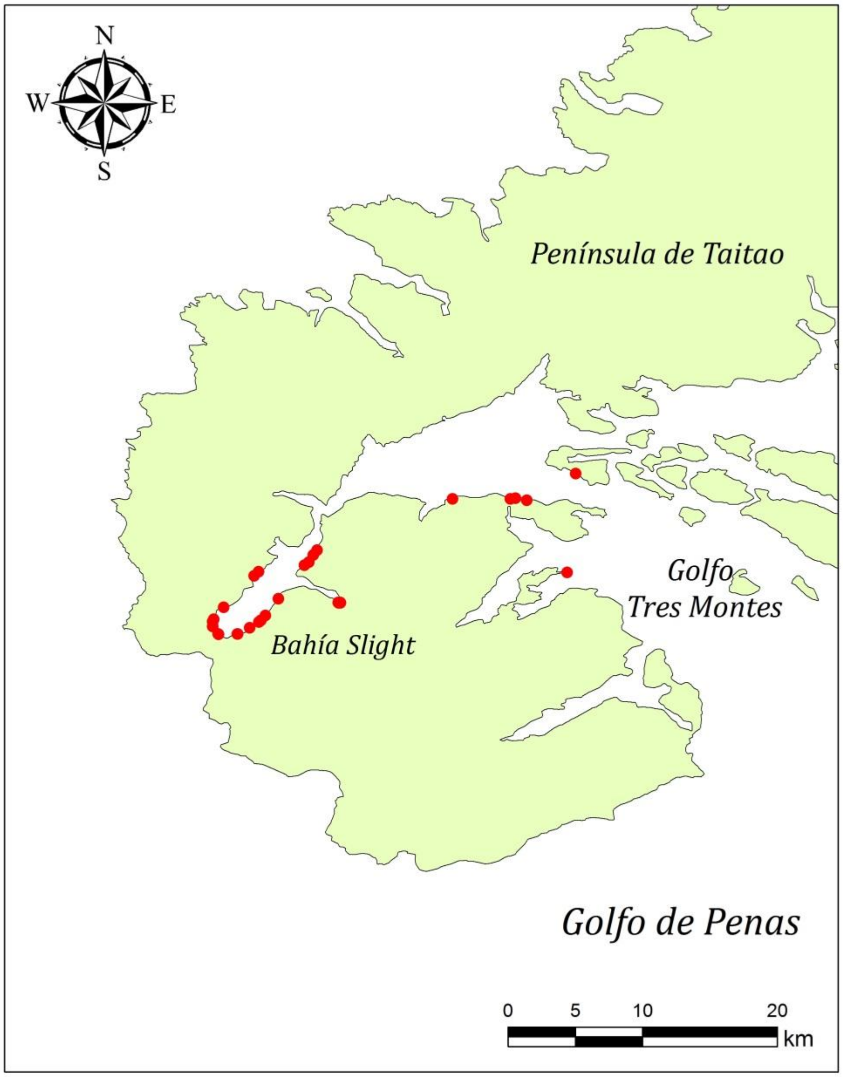

Figura 2. Ubicación geográfica de ballenas muertas, tanto del evento reciente como anteriores.

Figura 3 – Diagrama de rosa mostrando las orientaciones de las carcasas en el canal, expresado en grados, donde 0 es el Norte geográfico. Los números indican el numero de carcasas orientadas en cada cuadrante y el ovalo rojo indica el intervalo de confianza (95%) de la distribución de los datos.

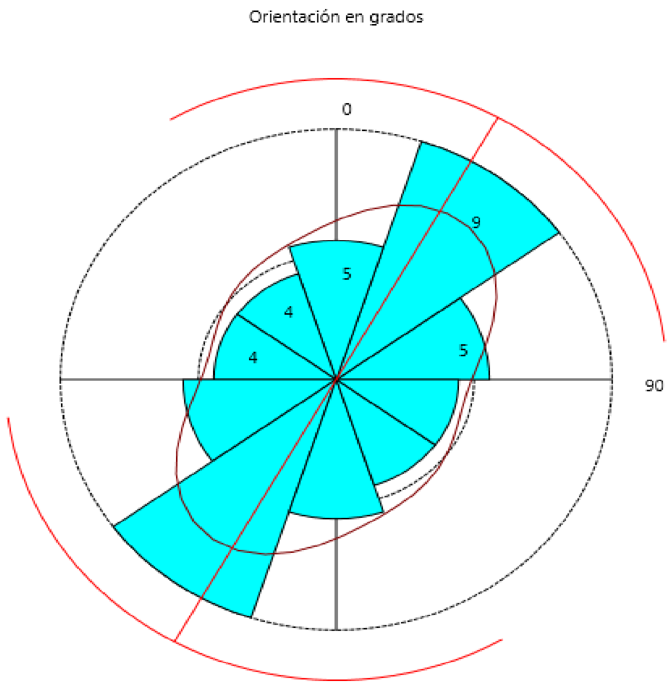

b) Especies

Se presume que la mayoría de los animales encontrados muertos son ballena sei (*B. borealis*). Sin embargo, la confirmación de especie directo en terreno solo se pudo hacer en aquellos cuerpos que presentaban un mejor estado de conservación y según las fotografías obtenidas. Adicionalmente, la corroboración taxonómica de 15 de 39 individuos muestreados fue realizada mediante análisis genéticos en el Laboratorio de Ecología Molecular de la Universidad de Chile. Para lo anterior se extrajo ADN de tejido (piel, músculo y/o encía de los individuos), se amplificó parte de la región control del ADN mitocondrial (ADNmt), región normalmente utilizada para la identificación de especies de cetáceos debido a que su acelerada tasa de mutación, lo que produce una alta variación, permitiendo la discriminación a nivel de especie (Ross et al 2003).

La amplificación de la región control (o D-loop) se realizó utilizando preferencialmente los partidores M13 Dlp1.5 y 8G (Dalebout et al. 2005) y secundariamente los partidores Dlp-10 y Dlp-5 (Baker et al 1993). Los productos de PCR fueron purificados y secuenciados en ambos sentidos en Macrogen, Corea. Las secuencias resultantes fueron alineadas y ensambladas manualmente mediante el programa Proseq 2.91 (Filatov, 2002). La identificación a nivel de especie se realizó

mediante el uso de dos programas de análisis comparativo de secuencias (1) “Basic Local Aligment Search tool (BLAST. www.blast.ncbi.nlm.nih.gov) y (2) DNA Survillance (Ross et al 2003; ver detalle de ambos programas en ANEXO II, Identificación Especie mediante análisis genéticos)

De la mayoría de las muestras de tejido recibidas (14 de 15 individuos) se obtuvo suficiente cantidad y calidad de ADN que permitió la amplificación de un fragmento de la región control de ADNmt de mayor tamaño (partidores M13 Dlp1.5 5’ y 8G Dalebout et al. 2005). Para el individuo restante se logró amplificar un fragmento de menor número de pares de bases descrito previamente (partidores Dlp-10 y Dlp-5, descrito Baker et al 1993).

Mediante el programa BLAST se obtuvo un 99% de confiabilidad que todas las secuencias analizadas y comparadas con esa base de datos correspondieron a ballena sei *Balaenoptera borealis* (Figura 1, ANEXO II – Identificación Especie mediante análisis genéticos). De la misma manera, mediante el programa DNA-Surveillance se obtuvo que la menor distancia genética (5%, Tabla 3), y por ende mayor semejanza entre las secuencias analizadas y las secuencias correspondientes a ballena sei, *Balaenoptera borealis.* Este resultado fue similar cuando se compararon las secuencias tanto a nivel de Orden Cetácea (Figura 2, ANEXO II – Identificación Especie mediante análisis genéticos) como a nivel de Suborden Misticeti (Figura 3).

Tabla 3. Distancias genéticas obtenidas en la comparación de una de las secuencias analizadas (seleccionada al azar como ejemplo) y la base de datos de DNA Surveillance. Se aprecia que la secuencia comparada tiene la menor distancia genética (0,05) (es decir, la mayor semejanza) con la especie *Balaenoptera borealis*

ID	Common Name	Distance
BborNA5.UA	Sei Whale	0.0501
BedeSA6.UA	Bryde's Whale (Common form)	0.0929
BedeJP1.HY	Bryde's Whale (Kochi form)	0.1066
BmusIC4.UA	Blue Whale	0.1631
BphyIC5.UA	Fin Whale	0.1696
ErobNEP397	null	0.1727
ErobWS03	Gray Whale	0.1729
MnovSEA9	Humpback Whale	0.1756
BacuNPSWFC	North Pacific Minke Whale	0.1771
BacuNA6.UA	North Atlantic Minke Whale	0.1794
BbonSO8.RH	Antartic Minke Whale	0.1855
CmarNZ1.SB	Pygmy Right Whale	0.1887
EglaNA9.SM	North Atlantic Right Whale	0.2126
CheBP03D	Hector's Dolphin	0.2333
EausSP16NP	Southern Right Whale	0.2384
BmysNEPSB1	Bowhead	0.2417
CcomL2.FP	Commerson's Dolphin	0.2443
LobsPE2.FC	Dusky Dolphin	0.2552
LacuNF6.FC	Atlantic White-Sided Dolphin	0.2554
KbreNZ01SP	Pygmy Sperm Whale	0.2573
TtruT2.JW	Bottlenose Dolphin (truncatus form)	0.2711
DcapCA1.PR	Common Dolphin (Long-Beaked)	0.272
DdelCA6.PR	Common Dolphin (Short-Beaked)	0.2788
GgriNZ1.MD	Risso's Dolphin	0.2788
LobINP8.FC	Pacific White-Sided Dolphin	0.283
BbaSW4965	Baird's Beaked Whale	0.2836
GmeINA8.LS	Long-Finned Pilot Whale	0.2949
PspiPE4.PR	Burmeister's Porpoise	0.295

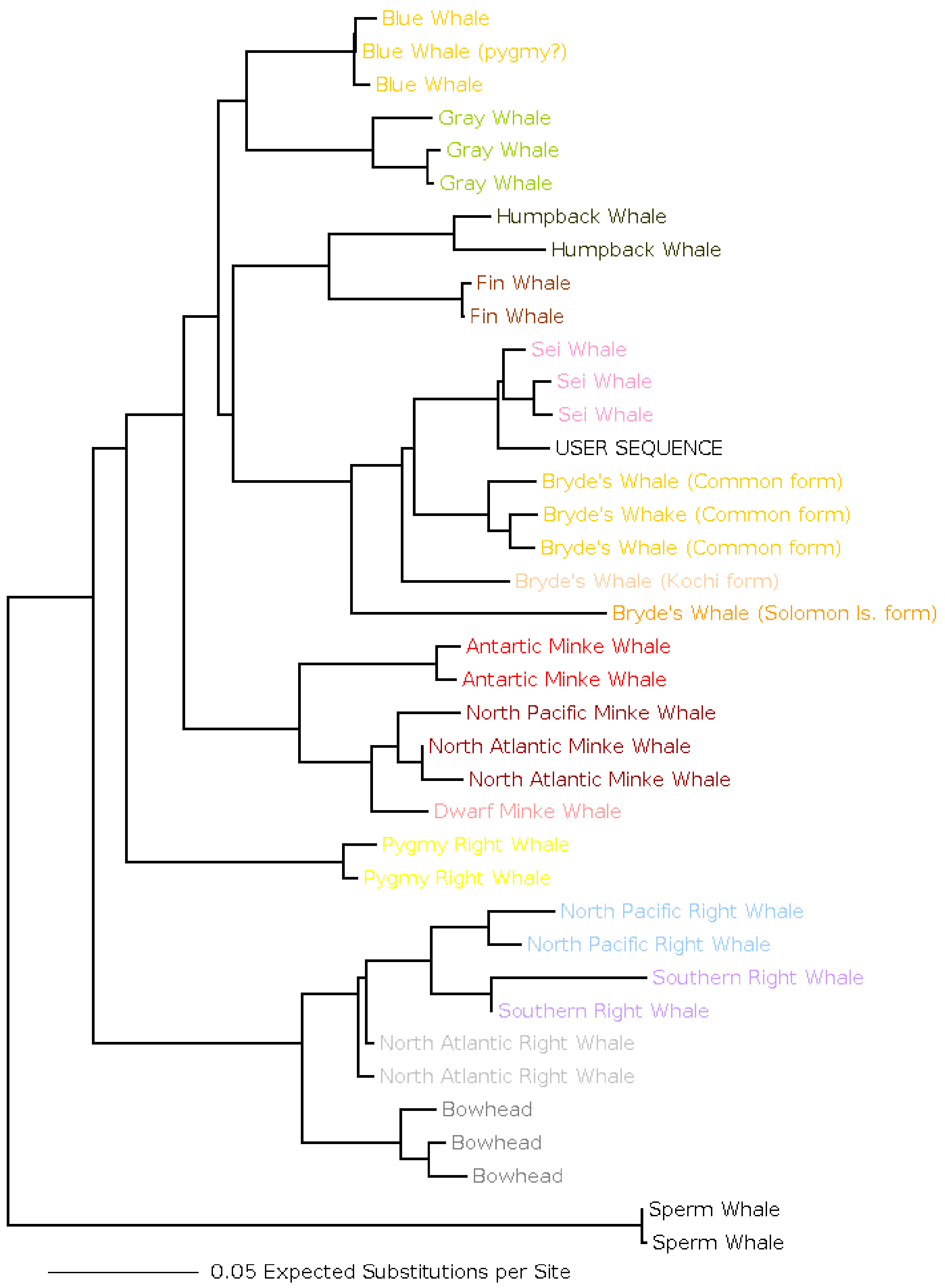

Figura 3. Ubicación de una de las secuencias analizada (ver flecha) en un árbol de distancia genética construido con información proveniente del orden suborden Misticeti en DNA Surveillance.

5) Análisis de microfracturas en el oído medio e interno

El análisis de parte de los huesos del oído medio e interno (apenas periótico) extraído del espécimen B8 no arrojó microfracturas en la lamina espiral (oído interno) y se observó que el huesecillo (estribo; oído medio) permanecía articulado a la ventana oval del periótico, **no mostrando signos de alteración que pudiesen haber sido provocadas por impactos mecánicos o acústicos**. De la misma manera, la región de los canales semi-circulares también se mostró anatómicamente intacta, no habiendo daño (Figura 4).

El hueso timpánico y por ende la articulación del martillo y la placa timpánica (equivalente al tímpano en misticetos) no pudo ser analizado, debido a que este elemento óseo, a pesar de requerido, no fue facilitado-trasladado a la Universidad de Chile para su análisis.

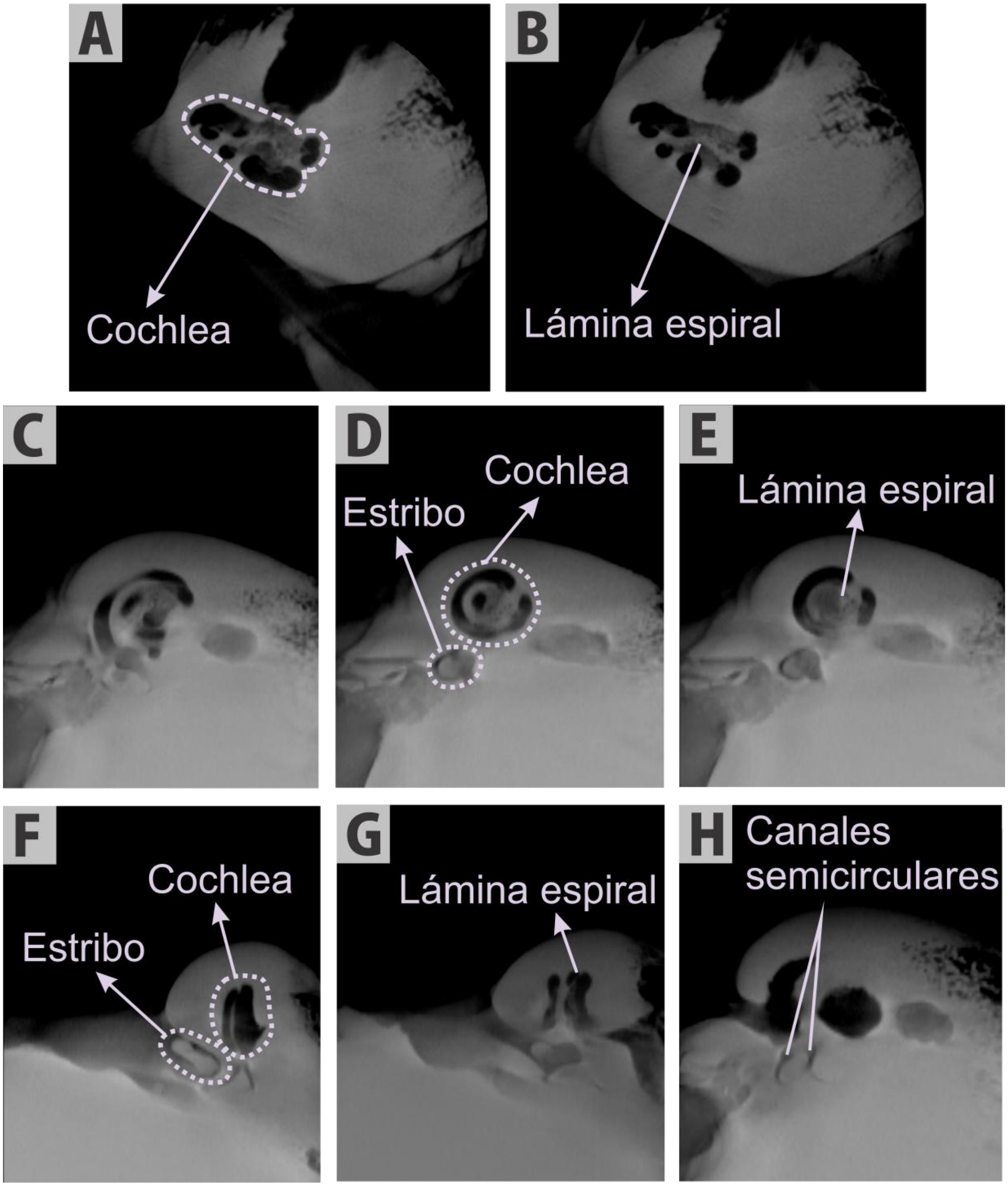

Figura 4. Cortes seleccionados de las imágenes obtenidas por tomografía volumétrica realizada en tomógrafo Morita (box de 60 mm, 500 cortes). Se observan en perfectas condiciones a todas las estructuras anatómicas acústico estructuralmente frágiles del oído medio (huesecillos: estribo) e interno (cochlea: lamina espiral), además de los canales semicirculares .

6) POTENCIALES CAUSAS DE MORTALIDAD DE BALLENAS

Según el diagnóstico en terreno y análisis de fotografías, **no se observa acción directa de actividades humanas, o de terceros** (heridas, traumas mecánicos evidentes, marcas de redes). Tampoco se observó evidente contaminación por hidrocarburos u otros químicos.

Por la posición de las ballenas en las playas (cuerpo posado sobre su parte dorsal), se asume que los animales murieron aún estando en el agua y llegaron por corrientes y vientos hasta las playas. Esto se debe a que los rorcuales luego de la muerte se hunden, debido al bajo contenido de grasa versus musculatura en comparación a los balénidos, por ejemplo, y luego del inicio de su descomposición se generan gases en la porción abdominal que hacen que las carcasas vuelvan a la superficie y permanezcan flotando con la porción ventral hacia arriba hasta que estos gases se liberen o hasta que varen en las playas. Por tanto en este informe nos referimos a mortalidad múltiple más que a varamiento.

Existen algunas características de esta mortalidad que hacen excluir algunas causas de muerte y potenciar a otras. Dado la ausencia de marcas de choques y o cortes y los resultados negativos de impacto acústico y mecánico en los análisis de tomografía de uno de los perióticos, se descarta la mortalidad por acción antropogénica (ej. impacto acústico o choque con embarcaciones). Además, la naturaleza múltiple del hallazgo hace poco probable cualquiera de las causas particulares, como choques por embarcaciones y-o muerte natural por senilidad. La presencia de un pinnípedo en el ensamble (resto esquelético muy fresco) hace poco probable la muerte por enfermedades. El posicionamiento de las carcasas (porción ventral hacia arriba) sugiere fuertemente un agente de muerte fuera del canal en donde fueron encontradas, es decir, alóctona.

La determinación de un agente de muerte alóctona, el número de individuos y la presencia de al menos un esqueleto reciente de otra categoria taxonomica (lobo marino de un pelo) en combinación con los resultados de los análisis de biotoxina, determina que la hipótesis que se evalúa con mayor fuerza es que la muerte de las ballenas se haya dado por la exposición y posterior intoxicación de los animales a toxinas generadas por afloramientos de fitoplancton (microalgas) nocivos. Los vectores (consumidores y filtradores) más probables de la biotoxina incluyen la sardina fueguina (*Sprattus fuegensis*) y/o langostino de los canales en su fase de larva pelágica (*Munida sp.*). Ambos vectores forman parte clave de la red trófica de los canales y fiordos patagónicos y por ende son un eslabón de gran importancia para las ballenas presentes en la región.

Para llegar a esta hipótesis y resultados, los análisis biotoxicológicos incluyeron:

- Un análisis cuantitativo de muestras de agua por microscopía para determinación de especies de fitoplancton.
- Pruebas cualitativas de Scotia Rapid Testing para detección de Veneno Paralizante de Mariscos (VPM) y Veneno Amnésico de Mariscos (VAM) en contenido estomacal.

- Pruebas cualitativas de Scotia Rapid Testing para detección de VPM y VAM en vectores de biotoxinas extraídos durante la expedición (almejas y choritos).
- Un análisis cualitativo por microscopía (presencia/ausencia) de contenido intestinal.

Los resultados que las muestras de agua arrojaron negativo para la presencia del fitoplancton esperado (normal después de 2-3 meses de las floraciones). Sin embargo, se encontró restos de toxina en los vectores y en los contenidos estomacales y restos de *Pseudonitzschia* en contenidos intestinales (Tabla 2).

Tabla 2. Resultados de pruebas biotoxicológicas

Prueba	Resultado
**Presencia de fitoplancton nocivo (muestras de agua)**	Negativo<br>(Todas las muestras de agua (6) resultaron negativas a la presencia de especies nocivas (Pseudonitzschia y Alexandrium))
**Toxina en vectores (muestras de almejas y choritos)**	2 positivos para VPM<br>2 negativos para VAM<br>(Muestras homogeneizadas de choritos (4 de Perumytilus purpuratus) de Bahía Slight y muestras de almejas (43 de varias especies) de las península Tres Montes, paso Holloway, ambas positivas para VPM, negativas a VAM)
**Toxina en contenido estomacal**	Positivo (ballena 8) para VAM<br>(Resultado de VAM positivo mayor a 20ug de ácido domóico por 100g de muestra)<br><br>Positivo (ballena 21) para VPM<br>(Resultado de VPM positivo pero en bajos niveles, estimación aproximada semicuantitativa de 60ug equivalentes de saxitoxina por 100 gramos de muestra)<br><br>1 ballena examinada por caso, una muestra de contenido estomacal examinada por ballena, un test de biotoxinas por muestra<br><br>Ballena 21 en Bahía buena, Ballena 8 en Bahía Slight
**Presencia contenido intestinal**	Positivo para *Pseudonitzschia*<br>(Positivo para ambas ballenas (#8 y #21), en mediana abundancia (escala relativa), muestras de contenido fecal y estomacal analizadas por ballena, ambas positivas)

7) CONCLUSION Y CONSIDERACIONES FINALES

La totalidad de los individuos analizados genéticamente correspondieron a la especie ballena sei *Balaenoptera boralis* (Tabla 4).

Tabla 4. Lista de ejemplares de códigos de muestras de ballenas varadas en el Golfo de Penas y su identificación especifica. En la columna especie se detalla que la totalidad de los individuos analizados genéticamente (15) correspondieron a la especie *Balaenoptera borealis*.

ID	Fecha	Familia	Especie
B1	27-05-2015	Balaenopteridae	
B2	27-05-2015	Balaenopteridae	
B3	27-05-2015	Balaenopteridae	*Balaenoptera borealis*
B4	27-05-2015	Balaenopteridae	
B5	27-05-2015	Balaenopteridae	
B6	27-05-2015	Balaenopteridae	*Balaenoptera borealis*
B7	27-05-2015	Balaenopteridae	
B8	27-05-2015	Balaenopteridae	*Balaenoptera borealis*
B9	27-05-2015	Balaenopteridae	
B10	27-05-2015	Balaenopteridae	*Balaenoptera borealis*
B11	27-05-2015	Balaenopteridae	*Balaenoptera borealis*
B12	27-05-2015	Balaenopteridae	
B13	27-05-2015	Balaenopteridae	*Balaenoptera borealis*
B14	27-05-2015	Balaenopteridae	*Balaenoptera borealis*
B15	27-05-2015	Balaenopteridae	*Balaenoptera borealis*
B16	27-05-2015	Balaenopteridae	*Balaenoptera borealis*
B17	27-05-2015	Balaenopteridae	*Balaenoptera borealis*
B18	27-05-2015	Balaenopteridae	
B19	27-05-2015	Balaenopteridae	*Balaenoptera borealis*
B20	27-05-2015	Balaenopteridae	
B21	27-05-2015	Balaenopteridae	*Balaenoptera borealis*
B22	27-05-2015	Balaenopteridae	*Balaenoptera borealis*
B23	27-05-2015	Balaenopteridae	*Balaenoptera borealis*
B24	27-05-2015	Balaenopteridae	*Balaenoptera borealis*
B25	27-05-2015	Balaenopteridae	
B26	28-05-2015	Balaenopteridae	
B27	28-05-2015	Balaenopteridae	
B28	29-05-2015	Balaenopteridae	
B29	29-05-2015	Balaenopteridae	
B30	29-05-2015	Balaenopteridae	
B31	29-05-2015	Balaenopteridae	
B32	29-05-2015	Balaenopteridae	
B33	29-05-2015	Balaenopteridae	
B34	29-05-2015	Balaenopteridae	
B35	29-05-2015	Balaenopteridae	
B36	29-05-2015	Balaenopteridae	
B37	29-05-2015	Balaenopteridae	
B38	29-05-2015	Balaenopteridae	
B39	29-05-2015	Balaenopteridae	

Los resultados obtenidos durante la expedición, sumado a la alta presencia de *Pseudonitzschia* durante febrero y marzo medidas en estaciones de monitoreo del programa de monitoreo de marea roja, administrado por la Sub Secretaría de Pesca y ejecutado por el Instituto de Fomento Pesquero, refuerzan la posibilidad de aceptación de la hipótesis de envenenamiento por biotoxinas por floraciones algales nocivas. No obstante, debido principalmente al mal estado de conservación de los animales que impidió una buena obtención de muestras (frescas) para su posterior análisis, y el tiempo limitado de permanencia en el área de estudio (1,5 días) para realizar un trabajo más exhaustivo, los resultados presentados aquí **no son concluyentes**. La presencia de toxinas en vectores en el área donde se encuentran las ballenas muertas o la presencia de toxinas en dos de las 29 ballenas encontradas con tejidos blandos a la vista, no puede ser considerado como una evidencia conclusiva, ni determinante. Las evidencias presentadas aquí son más bien agentes de construcción de hipótesis para ser evaluadas con mayor rigurosidad en el futuro cercano.

Por ejemplo, no se tiene seguridad debido al resultado no significativo del test para las orientaciones, de que el ensamble de las ballenas sea alóctona, es decir, que murieron fuera de la zona donde fueron encontradas, y principalmente no se pudo determinar a qué distancias estas pueden estar desde su punto de muerte (potencial área de intoxicación). Asimismo, el número de ballenas registradas en la expedición puede representar solo una fracción del número total de animales muertos. La compleja geomorfología de la costa, aislamiento y condiciones atmosféricas generaron condiciones desfavorables para la búsqueda de más animales. Esto sumado a que otros se puedan encontrar bajo la línea de marea y por tanto “invisibles” a una prospección marina como la ejecutada.

La mortalidad descrita en este informe evidencia un evento sin precedente en Chile y el mundo, en cuanto al número de individuos afectados. No obstante, la mortalidad de ballenas en la región no es un hecho nuevo. Al menos cuatro eventos se han registrado anteriormente que no fueron documentados en detalle. El más reciente, ocurrido entre enero y marzo de 2014 y documentado parcialmente por la profesional Sra. Carla Cristi, a través de fotografías y fichas resumidas de varamiento de fauna marina oficiales del Sernapesca, consta de 5 especímenes de ballenas, posiblemente de la especie Minke (*Balaenoptera acutorostrata*) o juveniles de otra especie de misticeto, dos en un canal interior de isla Kent, a cientos de metros de distancia entre sí, y las otras 3 bastante más al Sur, cerca del glaciar Bernardo, estas distribuidas a pocos metros de distancia entre sí. Estos documentos quedan a disposición de la investigación.

Por otro lado, la mortalidad masiva por intoxicación causada por floraciones algales nocivas ha sido descrita para otras dos especies de cetáceos alrededor del mundo, pero principalmente para las costas del Pacífico Nororiental (e.g. Geraci *et al*. 1989; Durbin *et al*. 2002; Doucette *et al*. 2006). Este hecho es de gran importancia por el efecto que pudiera tener a niveles poblaciones, particularmente para aquellas especies que presentan un estado de conservación amenazado (vulnerables o en peligro, como es el caso de variadas especies de ballenas).

Es por esto que la formalización de un protocolo de respuesta a varamientos de mamíferos marinos no es sólo importante para la estandarización de metodologías y la recolección de información clave, sino que además es de gran valor para entender las causas de muerte, sean naturales o por acciones humanas. Así mismo, una buena planificación y acción tiene gran relevancia para las agencias coordinadoras con el fin que dichos muestreos se hagan de forma responsable, eficiente y ordenada, y que los responsables por obtener dicha información tengan la experiencia y respaldo científico que merece dicho trabajo.

Anexos

Anexo I

INFORME DE LAS NECROPSIAS PARCIALES REALIZADAS EN CUATRO CADÁVERES DE BALLENAS BOBAS (*Balaenoptera borealis*, “Sei whale”) EN BAHÍA SLIGHT, NOROESTE DEL GOLFO DE PENAS, XI REGIÓN

Por

G. Garrido, B. Cáceres, C. Calderón, T. García, J. Acevedo, D. Haro y A. Aguayo Lobo
Grupo de Punta Arenas

Introducción

El objetivo del presente informe (Anexo), es mostrar si hubo o no evidencias de interacciones humanas en la muerte de cuatro ejemplares de ballena boba o “Sei whale”, en las aguas del Golfo de Penas, XI región, que fueron examinadas durante los días 27, 28 y 29 de mayo de 2015; después de alrededor de 30 días de haber sido observadas por personal de la Estación Científica de Huinay.

Por otra parte, estimamos necesario hacer notar, que el objetivo de una necropsia completa es conocer la causa de muerte de un organismo. En consecuencia, nos parece necesario diferenciar dos Protocolos: El primero, el que busca la causa de muerte (Protocolo de Necropsia) y el segundo, el que busca signos de interacciones humanas en la muerte de las ballenas (Protocolo del Ilícito ambiental). Esta diferencia es fundamental para poder diferenciar las causas de muerte o las causas de los varamientos de los cetáceos; pues se acepta como varamiento de cetáceos, cuando uno o más ejemplares arriban vivos a la costa y posteriormente, mueren en tierra (Perrin y Geraci, 2002; Aguayo-Lobo, 2013; Haro *et al.* 2015).

Desarrollo de las necropsias

De los cadáveres de **18** ejemplares completos de ballenas del género *Balaenoptera*, se lograron medir **16** ejemplares y sexar sólo a **12**. De estos últimos, **6** corresponden a machos (Ejemplares 2, 10, 17, 21, 23 y 34) y **5** a hembras (Ejemplar 3, 11, 12, 15 y 22) y **1** de sexo desconocido (Ejemplar 8). De estos **11** ejemplares sexados, se pudo realizar el examen externo, para conocer si hubo o no ilícito ambiental, y las necropsias parciales o incompletas, con el objeto de intentar conocer la causa de muerte de cuatro cadáveres; ejemplares 8, 21, 22 y 23. Se registraron además **12** cráneos, pertenecientes al género *Balaenoptera*, de los cuales se midieron **9**, con excepción de **3** ejemplares (13, 18 y 27). Debido a las dificultades topográficas del terreno, no se tuvo acceso a **9** ejemplares (28, 29, 30, 31, 32, 36, 37, 38 y 39).

Ejemplares examinados y muestreados

1. **Ejemplar N° 8: Género *Balaenoptera***

El animal examinado corresponde a un ejemplar de *Balaenoptera borealis*, considerando que algunas medidas corporales como la longitud de los surcos toraco-ventrales, en relación a la longitud total, no alcanzan el ombligo, siendo esta una de las características diagnósticas de esta especie. Además, la forma, color y características de los flecos de las barbas, confirmar la identificación de esta especie (Figura 1). El estudio genético de algunas muestras están siendo analizadas en la Facultad de Ciencias de la Universidad de Chile cuyos resultados esperamos confirmen la determinación de la especie.

Examen externo

Al examen externo del ejemplar número 8, de 15,27 metros de longitud total (medida aproximada debido a la torsión del pedúnculo caudal); sexo desconocido, debido a la avanzada descomposición de la zona genital (Figura 2). La piel se encontraba de un tono blanco anaranjado, indicando la pérdida parcial o total de epidermis por zonas, sin evidencias de heridas recientes o cicatrices antiguas, ni de traumas, tumores, inflamaciones o parásitos externos, ni tampoco evidencia de redes o mallas que pudieron haber gatillado el varamiento y posterior muerte del ejemplar, o que el ejemplar murió en altamar y fue llevado, en esa condición, a la deriva por las corrientes prevalecientes en el Golfo de Penas.

Tomando en cuenta que el animal se encontraba de cúbito lateral derecho sobre una playa arenosa de grano fino, no se pudo observar el costado derecho del mismo. En consecuencia, el costado izquierdo, el dorso y el vientre del animal; es decir, todas las partes del cuerpo examinadas, no mostraban signos de lesiones antropogénicas (Figura 3).

Cabe mencionar que tampoco se encontraron restos de hidrocarburos, sustancias oleosas ni otras sustancias químicas nocivas cercanas al ejemplar.

Necropsia

Dado el estado de descomposición del ejemplar y las dificultades técnicas para manipular un animal de tales dimensiones, sólo se pudo practicar una necropsia parcial. Se realizó una incisión desde la parte posterior del tórax hasta la parte anterior del abdomen (Figura 4), obteniendo luego, muestras de piel, grasa, músculo, costillas, pared estomacal, contenido estomacal, contenido rectal y barbas desprendidas de la boca, para posteriores análisis de laboratorio. Las muestras de contenido estomacal y contenido rectal obtenidas podrían servir para el análisis de posible intoxicación por marea roja, investigación que está llevando a cabo el Dr. David Cassis. Al retirar la capa muscular de la zona torácica izquierda, se observó una sección de musculatura de coloración más oscura, con acumulación de coágulos sanguíneos por gravedad (Figuras 5 a y b), evidenciando que existió un trauma, el cual puede haberse presentado *pre o post mortem*. No se observó esófago, tráquea, corazón en la cavidad torácica; o hígado e intestino en el abdomen, sin poder examinar la cavidad abdominal y pélvica en forma exhaustiva.

En cambio, sí se tuvo éxito en realizar una incisión cercana a la articulación mandibular, con el objeto de extraer la bula tímpano-periótica. Lamentablemente, no fue posible obtener el complejo auditivo completo.

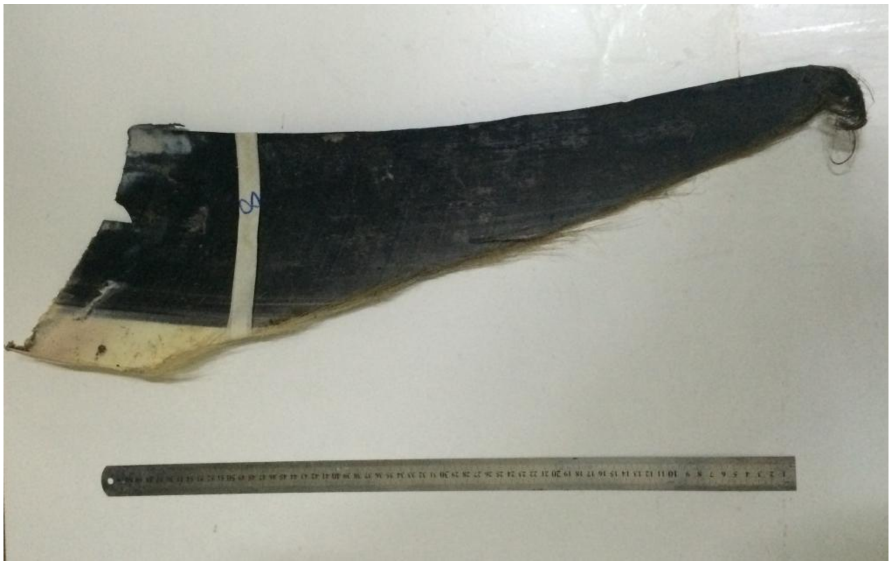

Figura 1. Barba correspondiente a animal número 8, de una longitud total de 15.27 m.

La barba medía una longitud de 56 cm de largo y 25 cm de ancho. Fotografía: Gabriela Garrido.

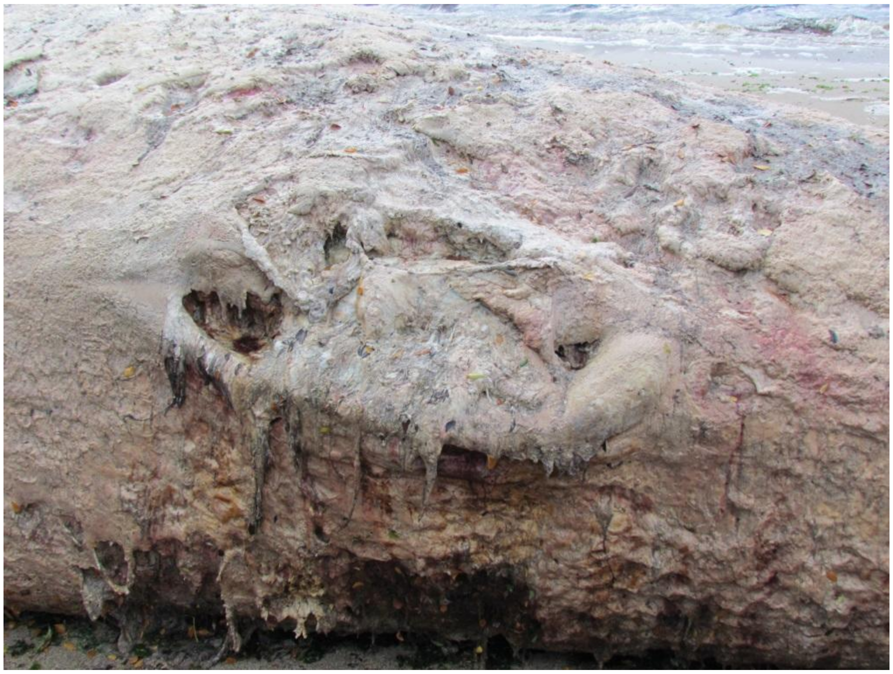

Figura 2. Fotografía zona genital de individuo número 8, donde se evidencia el avanzado estado de descomposición de la zona genital. Fotografía: Natalia Toledo.

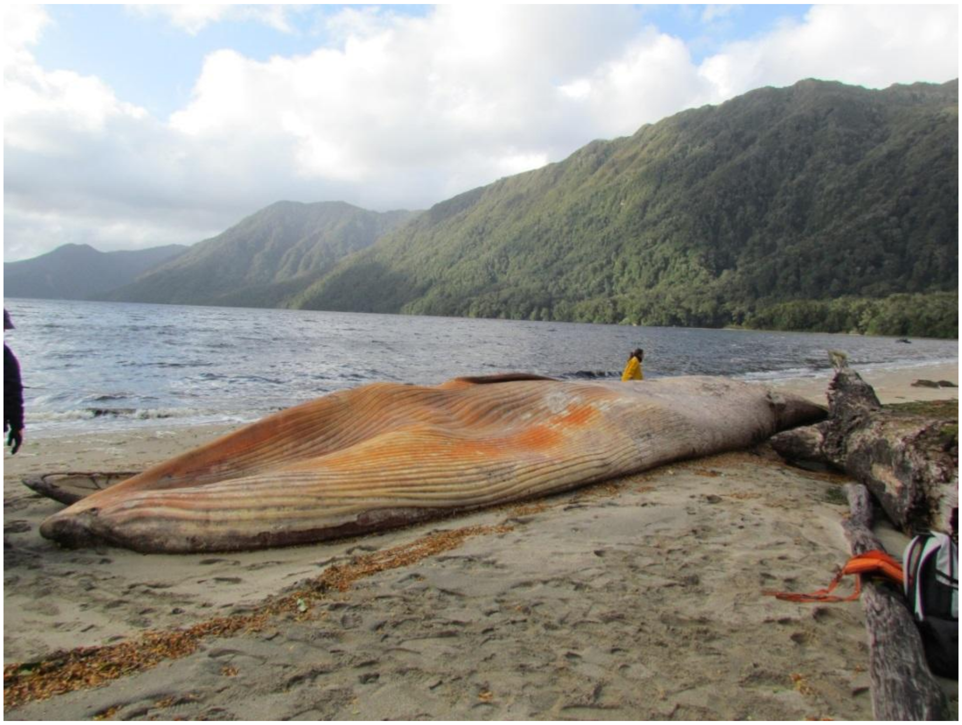

Figura 3. Ejemplar número 8, en posición de cúbito lateral derecho, donde se observa la longitud de los pliegues toraco-ventrales. Fotografía: Nicolás Leiva.

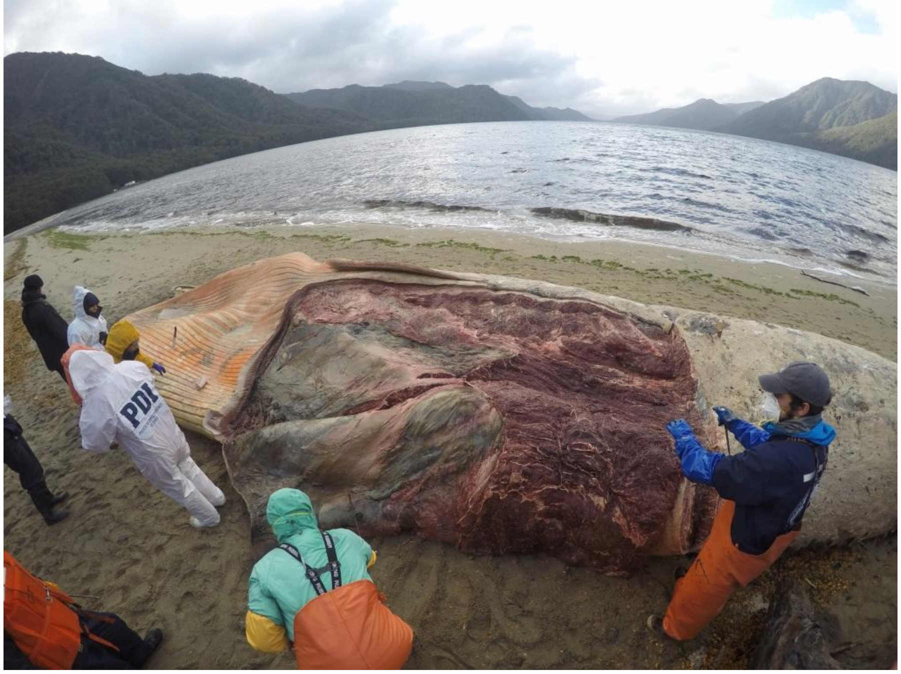

Figura 4. Incisión toraco-abdominal realizada al ejemplar número 8. Fotografía: Nicolás Leiva.

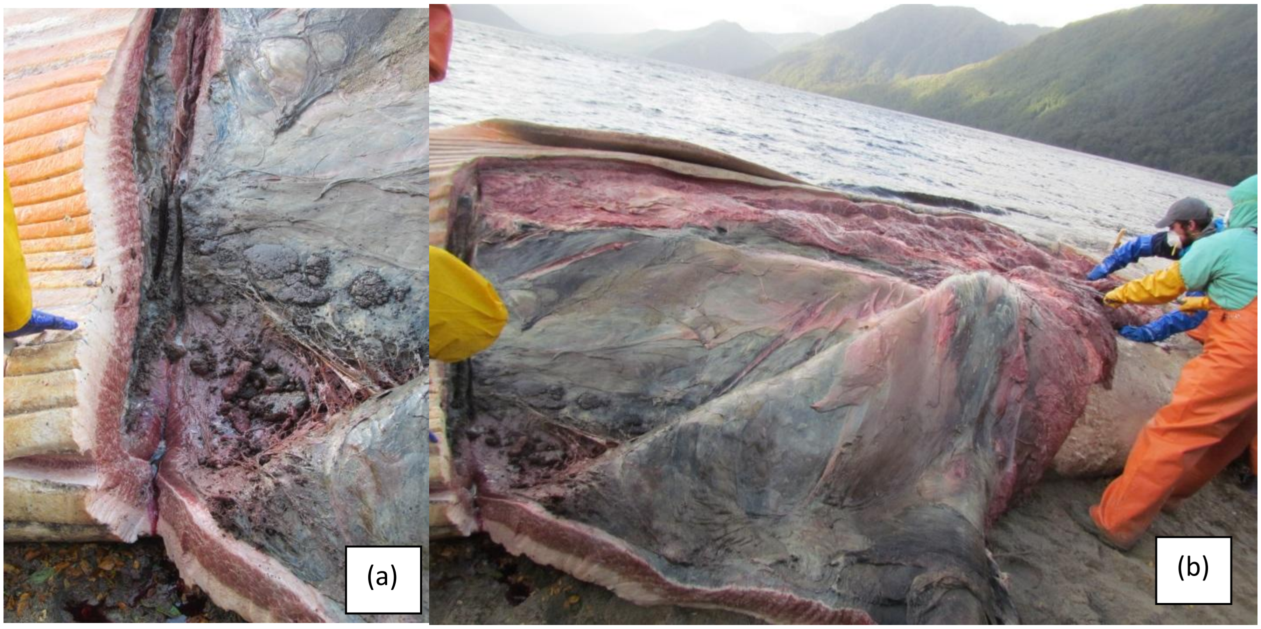

Figura 5. (a) Vista a formación de coágulos acumulados en zona ventral por acción de gravedad. (b) Vista de hematoma observado en la zona torácica. Fotografía: Natalia Toledo.

Personal participante en la necropsia: Gabriela Garrido y Benjamín Cáceres, Museo de Historia Natural Río Seco; Jorge Acevedo, Fundación CEQUA; Camila Calderón, Universidad San Sebastián, Concepción; Trinidad García, Universidad de Concepción; Mauricio Ulloa y Marisol Romero, SERNAPESCA.

2. Ejemplar N° 21: Género *Balaenoptera*

Al igual que el ejemplar anterior (N°8), basado en las mismas características diagnosticas se ha determinado que el ejemplar corresponde a la especie *Balaenoptera borealis* (Figura 6 y 7), a la espera de la confirmación a través del estudio genético.

Examen externo

Al examen externo del ejemplar 21, de 8,3 metros de largo y sexo macho. Este ejemplar presentaba coloración semejante a la anterior blanco anaranjado (Figura 8), indicando una pérdida parcial o completa de la epidermis. No hay evidencias de heridas recientes o cicatrices antiguas, ni de traumas, tumores, inflamaciones o parásitos externos, ni de redes o mallas de pesca que puedan asociarse a la muerte de este animal. Tomando en cuenta que el animal se encontraba de cúbito lateral izquierdo en un sustrato rocoso y parcialmente cubierto por vegetación, no se

pudieron observar signos o síntomas de terceros ni en el vientre, ni en la cabeza, ni en la cola o en el costado derecho del animal. Cabe mencionar que no se encontraron restos de hidrocarburos, sustancias oleosas ni otras sustancias químicas nocivas cercanas al ejemplar.

Necropsia

Para la obtención de muestras y tener acceso a las vísceras internas se realizó una incisión que abarcó la parte posterior del tórax hasta la parte anterior del abdomen (Figura 9). Se obtuvieron muestras de piel, grasa, músculo, contenido estomacal y barbas desprendidas de la boca. No se pudo observar esófago, tráquea, corazón, baso, riñones, hígado ni intestino. Las muestras obtenidas de contenido estomacal, podrían servir para posterior análisis de presencia de células o toxinas que indiquen intoxicación por marea roja, estudios de responsabilidad del Dr. David Cassis.

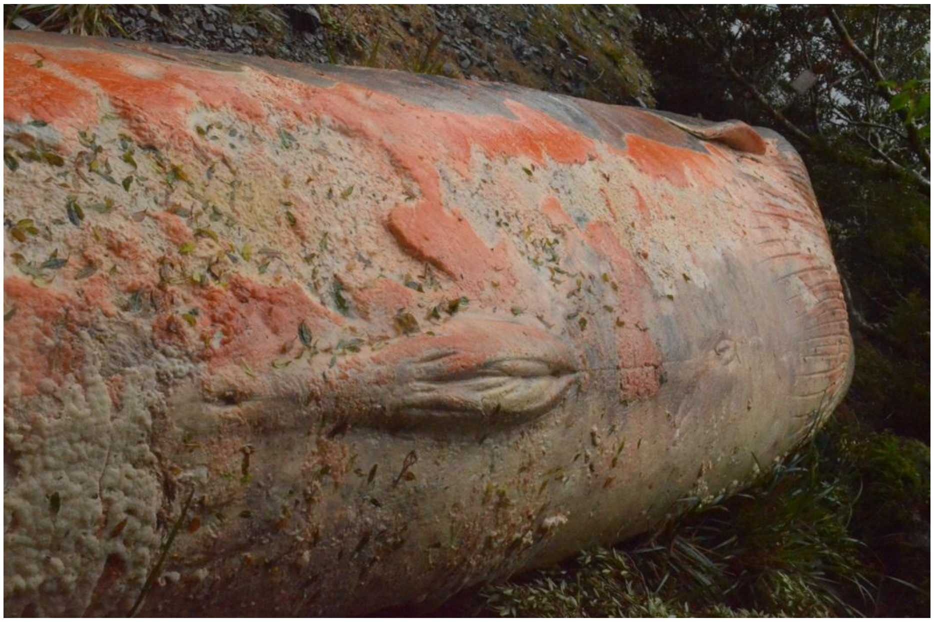

Figura 6. Fotografía muestra claramente el fin de los surcos toraco-ventrales anterior al ombligo.

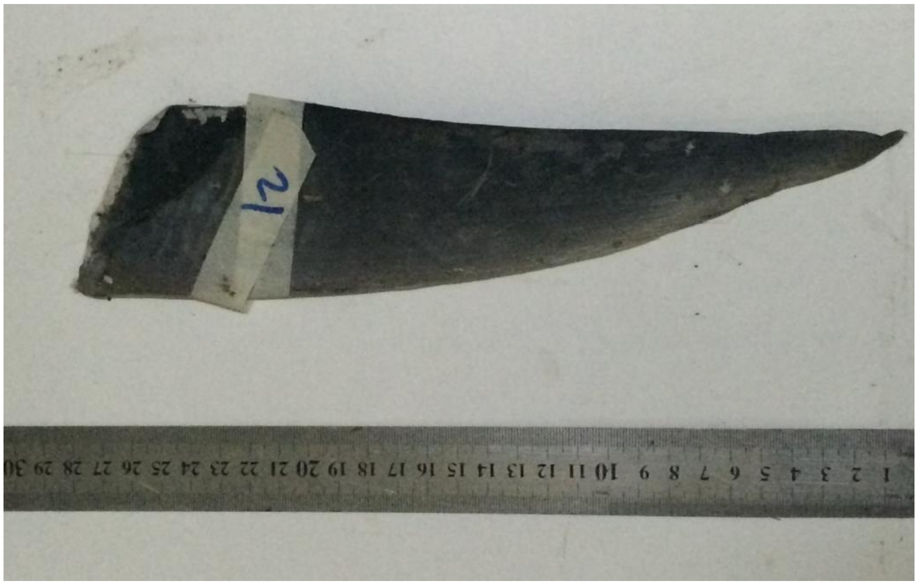

Fotografía 7. Barba individuo número 21 de longitud total: 8,3 m.

Longitud barba: 20 cm, ancho barba: 7 cm.

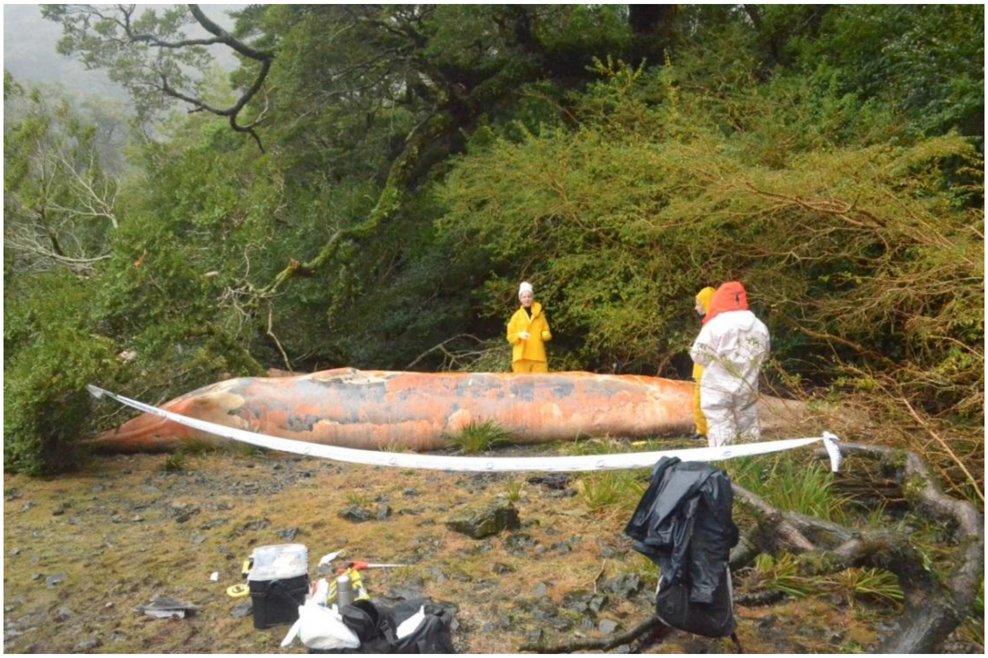

Figura 8. Ejemplar número 21 en posición de cúbito lateral izquierdo. Fotografía: Henry Gohlich.

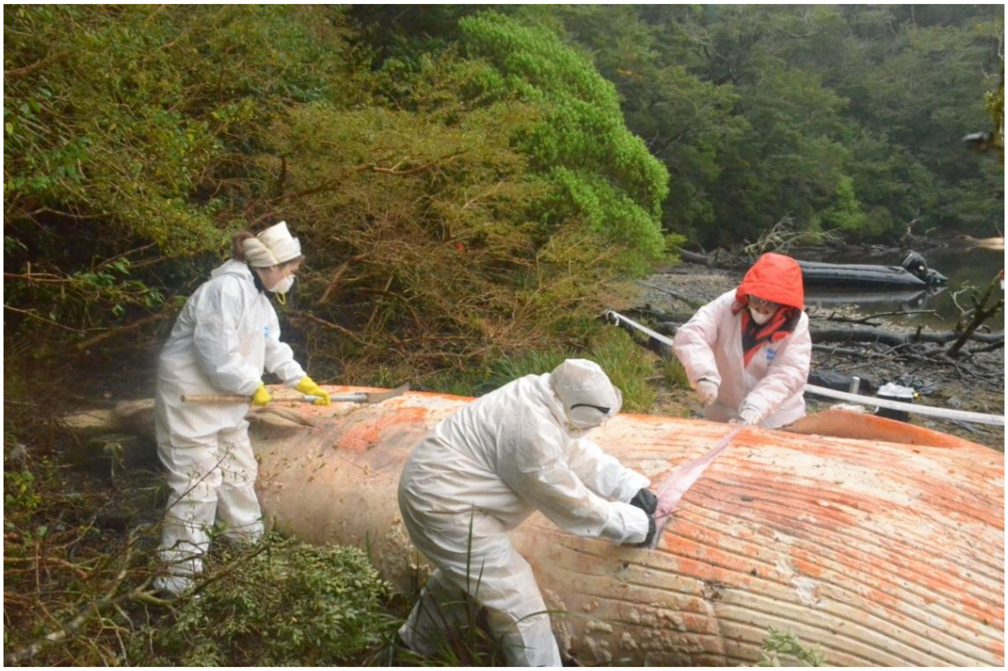

Figura 9. La incisión realizada en la necropsia y para obtención de muestras se realizó desde la parte posterior del tórax hasta la parte anterior del abdomen. Fotografía: Henry Gohlich.

Personal participante en la necropsia: Gabriela Garrido y Benjamín Cáceres, Museo de Historia Natural Río Seco; Jorge Acevedo, Fundación CEQUA; Camila Calderón, Universidad San Sebastián, Concepción; Trinidad García, Universidad de Concepción; Henry Gohlich, Fundación Huinay, Natalia Toledo y Marisol Romero, SERNAPESCA.

3. **Ejemplar N° 22: Género *Balaenoptera***

Al igual que los ejemplares anteriores (N°8 y 21), este corresponde a la especie *Balaenoptera borealis*, basado en las mismas características diagnósticas ya mencionadas (Figura 10); esperando el resultado de los estudios genéticos que confirmen la determinación de especie.

Examen externo

Al examen externo del ejemplar 22, de 10 metros de largo, sexo hembra. La piel presentaba una coloración blanco anaranjada, indicando la pérdida parcial o completa de la epidermis. En la región abdominal se observó un estado avanzado de descomposición y una gran pérdida de tejido muscular, debido a la acción de aves carroñeras observadas en el lugar (Figura 11 y 12). No hay evidencias de heridas recientes o cicatrices antiguas, ni de traumas, tumores, inflamaciones o parásitos externos, ni de redes o mallas de pesca que puedan asociarse a la muerte de este animal.

Tomando en cuenta que el animal se encontraba de cúbito lateral izquierdo en un sustrato rocoso y con presencia de vegetación, no se pudieron observar signos o lesiones provocados por la acción de terceros en el costado izquierdo del mismo.

Cabe mencionar que no se encontraron restos de hidrocarburos, sustancias oleosas ni otras sustancias químicas nocivas cercanas al ejemplar.

Necropsia

Se realizó una incisión desde la parte anterior del abdomen (Figura 13), hasta la parte anterior de una gran herida producida por animales carroñeros. Luego, se realizó una incisión en la zona rectal. Posteriormente se obtuvieron muestras de piel, músculo, contenido estomacal, intestino, contenido intestinal, parásitos intestinales y riñones. No se obtuvieron muestras de esófago, hígado, corazón, baso, útero ni gónadas. Las muestras de contenido estomacal y contenido intestinal, están siendo analizadas para determinar de presencia o ausencia de células o toxinas de fitoplanctónicas, investigación a cargo del Dr. David Cassis.

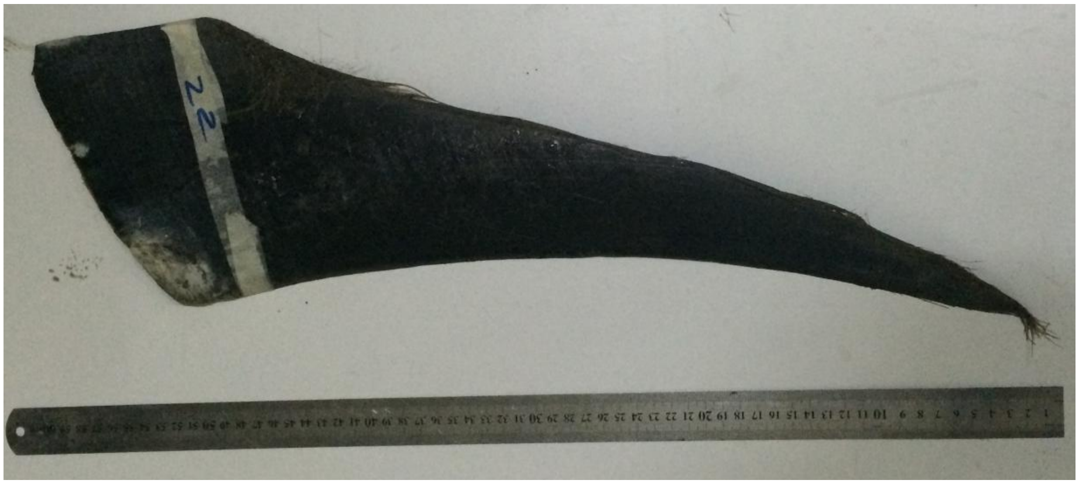

Figura 10. Barba perteneciente a ejemplar número 22 de 10 m de longitud.

Longitud barba: 43 cm y ancho: 18 cm. Fotografía: Gabriela Garrido.

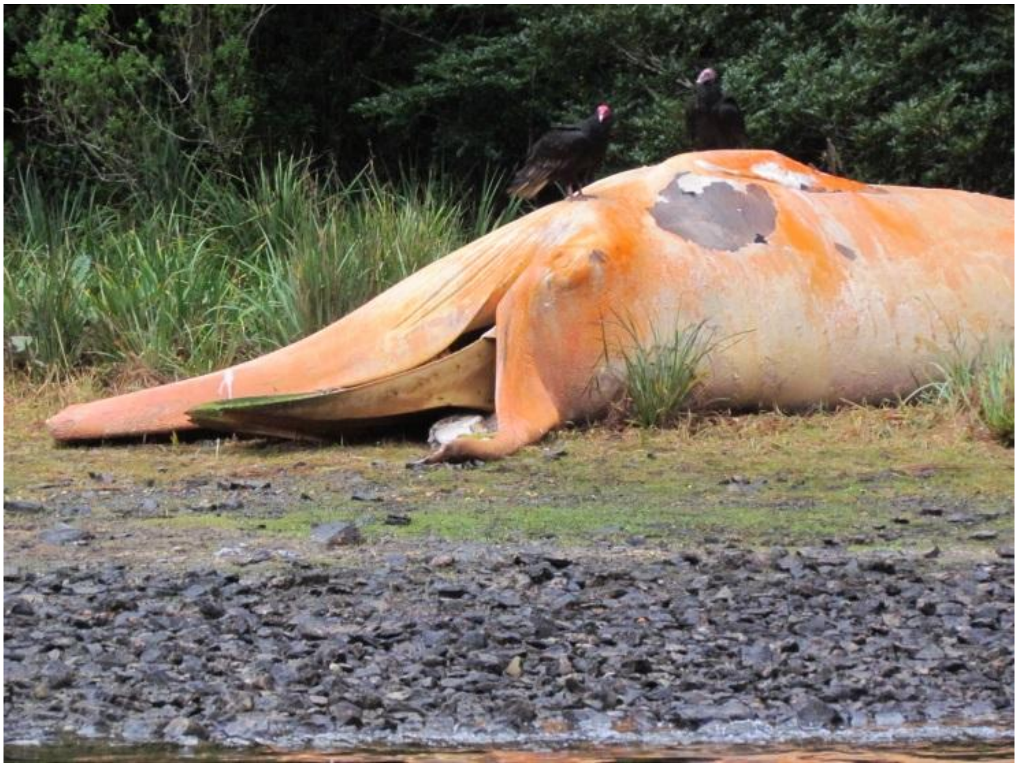

Figura 11. Posición de cúbito lateral izquierdo, evidencia de aves carroñeras en el área. Fotografías: Natalia Toledo.

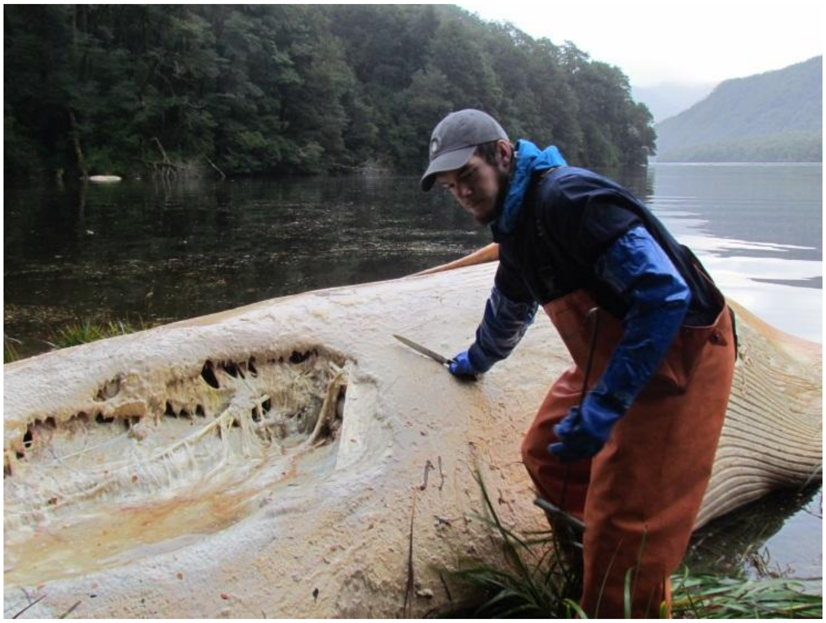

Figura 12. Fotografía evidencia el alto grado de descomposición en la zona abdominal del ejemplar 22. Fotografías: Natalia Toledo.

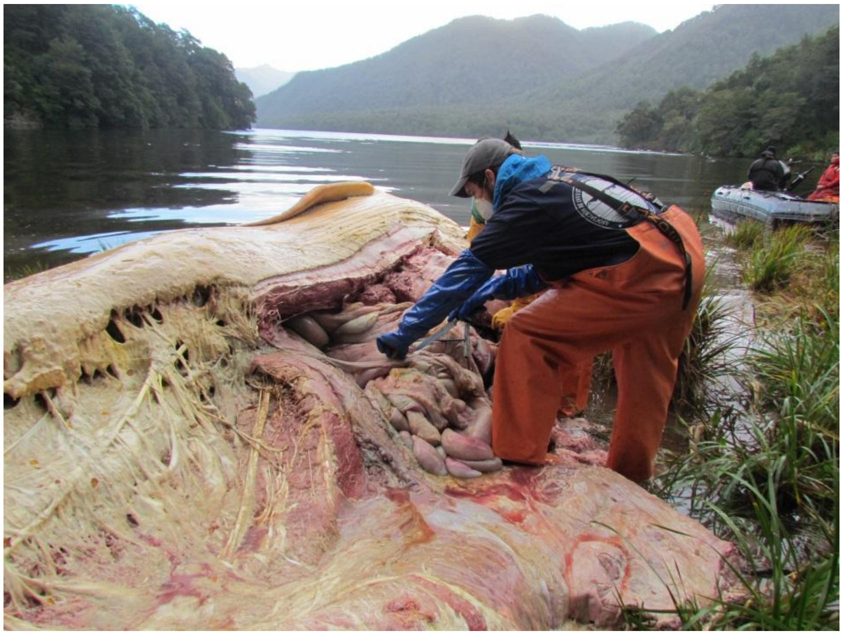

Figura 13. Incisión abdominal del ejemplar Número 22. Fotografía: Natalia Toledo.

Personal participante en la necropsia: Gabriela Garrido y Benjamín Cáceres, Museo de Historia Natural Río Seco; Jorge Acevedo, Fundación CEQUA; Nicolás Soto y Natalia Toledo, SERNAPESCA.

4. **Ejemplar Número 23: Género *Balaenoptera***

Al igual que los ejemplares anteriores (N°8, 21 y 22), este corresponde a la especie *Balaenoptera borealis*, basado en las mismas evidencias mostradas anteriormente (Figura 14). Así mismo, se espera la confirmación genética.

Examen externo

Al examen externo, el ejemplar 23, de 11,5 metros de largo y sexo macho. La piel presentaba una coloración semejante a las anteriores, indicando posiblemente al menos uno o dos meses de muerte. No existen evidencias de traumas, tumores, inflamaciones o parásitos externos, ni de redes o mallas de pesca que puedan asociarse a la muerte de este animal. Tomando en cuenta que el ejemplar se encontraba en posición cúbito lateral derecho (Figura 15), sobre sobre una playa rocosa con vegetación parcial y expuesta a la marea, no se pudo observar el costado derecho del mismo. En consecuencia, no es posible afirmar de forma categórica que no hay lesiones externas atribuibles a acciones antropogénicas en el costado izquierdo del mismo.

Tampoco se encontraron restos de hidrocarburos, sustancias oleosas ni otras sustancias químicas nocivas cercanas al ejemplar.

Necropsia

Se realizó una incisión desde la parte posterior del tórax hasta la parte anterior de la pelvis (Figura 16). Luego se obtuvieron muestras de piel, grasa, músculo, estómago y contenido estomacal. Además se recolectaron barbas adyacentes; sin embargo, no es posible determinar con certeza si pertenecen al ejemplar 23. No se inspeccionaron el corazón, hígado, baso, páncreas, riñones ni gónadas. Las muestras de contenido estomacal están siendo analizadas por el Dr. David Cassis.

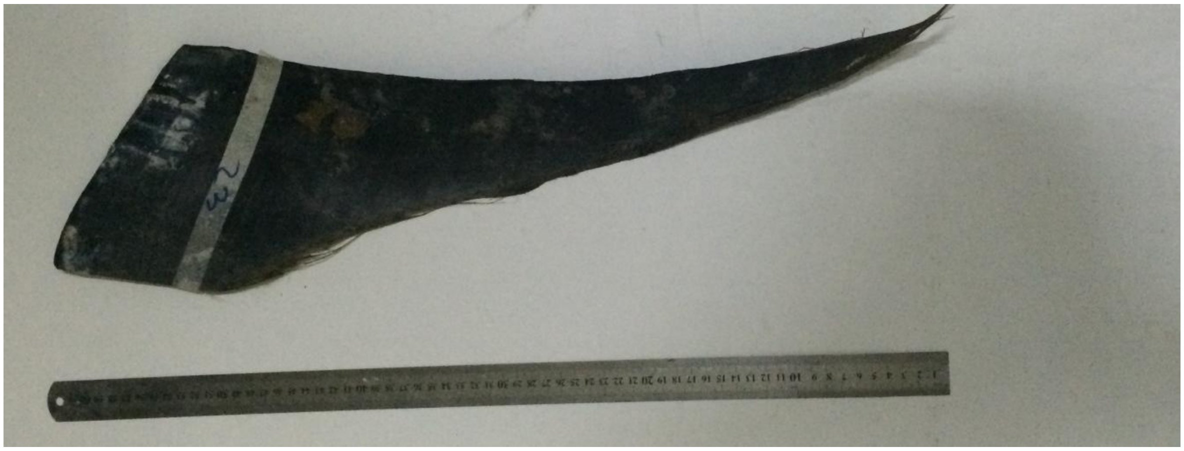

Figura 14. Barba de ejemplar número 23, de longitud total: 11,5 m.

Longitud barba: 41 cm y ancho: 18,6 cm.

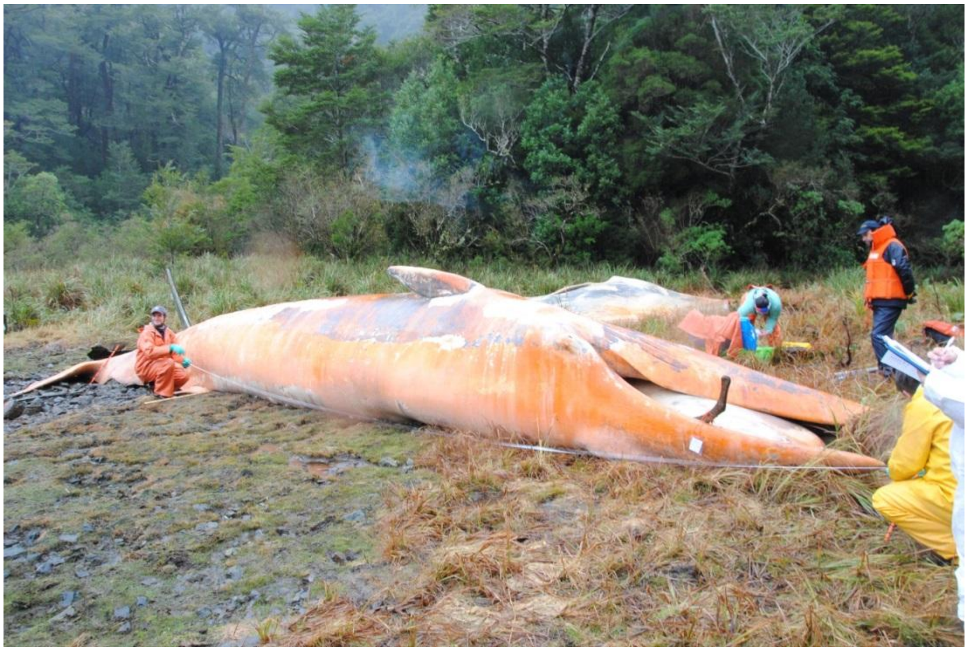

Figura 15. Ejemplar Número 23 en posición de cúbito lateral derecho. Fotografía: Directemar, Aysén.

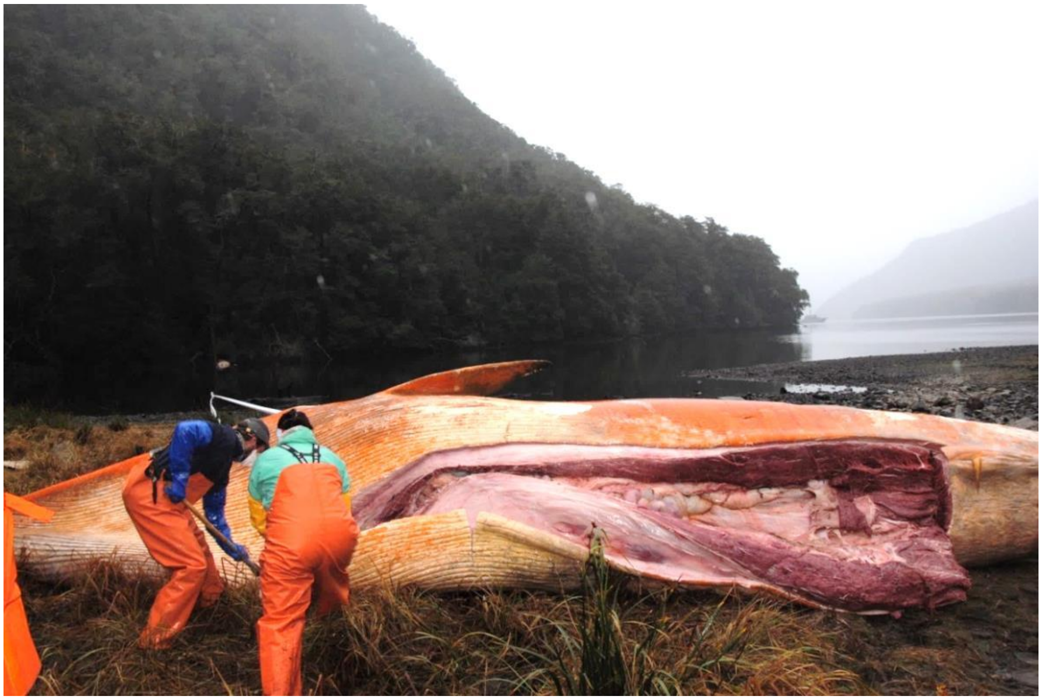

Figura 16. Incisión toraco-abdominal del ejemplar 23. Fotografía: Directemar, Aysén.

Personal participante en la necropsia: Gabriela Garrido y Benjamín Cáceres, Museo de Historia Natural Río Seco y Jorge Acevedo, Fundación CEQUA.

Conclusiones

1) Las medidas morfométricas, tales como la longitud de los surcos toraco-ventrales y el examen de las características diagnósticas de las barbas de los cuatro ejemplares estudiados, nos permiten inferir que todos pertenecen a la misma especie: *Balaenoptera borealis,* Lesson 1828. Esperando la confirmación genética de esta determinación.

2) Según las evidencias colectadas en terreno, bahía Slight y en caleta Buena. Para los cuatro ejemplares muestreados, es posible afirmar que no se encontraron lesiones, traumas, cicatrices, heridas o inflamaciones, que puedan atribuirse a acciones antropogénicas y que éstas hayan contribuido a la muerte de los ejemplares. Además, en el mismo sentido no se encontraron redes, mallas o aparejos de pesca en los cadáveres de los animales y tampoco, se encontraron restos de hidrocarburos, sustancias oleosas, ni otras sustancias químicas nocivas cercanas, que pudieron haber causado el varamiento y posterior muerte de los cuatro ejemplares examinados. En consecuencia, no habría existido ilícito ambiental en esta mortalidad.

3) Debido a las dificultades técnicas y topográficas de la región, a la falta de personal con experiencia en necropsias de mamíferos marinos y junto con, el tiempo limitado de trabajo en el área de estudio, sólo fue posible realizar cuatro necropsias parciales, de los 16 ejemplares medidos y, muestreados superficialmente.

4) Considerando el diferente estado de descomposición de los cadáveres completos (18) y de los cráneos registrados (12) de las ballenas, se puede inferir que los eventos de muerte de *Balaenoptera borealis* en el área de estudio, corresponderían por lo menos a tres durante los últimos 2 años.

Agradecimientos

Deseamos manifestar nuestros agradecimientos al Dr. Mauricio Ulloa, al Dr. Rodrigo Hucke, por la gentil invitación para participar de esta expedición; como así mismo agradecemos el apoyo brindado por la Armada de Chile, por haber facilitado el patrullero P.S.G Ortiz al mando del capitán José Cabezas. También agradecemos a los colegas de SERNAPESCA, FUNDACION HUINAY, PDI y a la Dr. María José Pérez por la esperada ratificación de la determinación de la especie.

Literatura Citada

Perrin W.F y J. R Geraci. 2002. Stranding. Pp 1192-1197. En: W. F Perrin, B. Wursig y J.G.M Thewisen (Eds). Encycopedia of Marine Mammals. Academic Press. California.

Aguayo Lobo, A. 2013. Varamiento de los Cetáceos. Primer Simposio de Investigación de Mamíferos Marinos de Chile. Panthalasa. Facultad de Medicina Veterinaria Universidad Mayor. Santiago, 8-10 Agosto de 2013. Trabajo no publicado. 6 páginas.

Daniela Haro, Anelio Aguayo-Lobo, Olivia Blank, Constanza Cifuentes, Catherine Dougnac, Cristobal Arredondo, Catalina Pardo e Iris Cáceres-Sáez. 2015. Nuevo varamiento masivo de orca falsa, *Pseudorca crassidens*, en el Estrecho de Magallanes, Chile. Revista de Biología Marina y Oceanografía. Vol 50. N°1: 149-155.

Punta Arenas, 30 de Julio de 2015

Anexo II

INFORME DE LA IDENTIFICACION A NIVEL DE ESPECIE DE LAS BALLENAS VARADAS MASIVAMENTE EN BAHÍA SLIGHT, NOROESTE DEL GOLFO DE PENAS, XI REGIÓN, MEDIANTE TÉCNICAS MOLECULARES

Dr M.José Pérez-Alvarez
Investigador Postdoctoral
Instituto de Ecología y Biodiversidad
Facultad de Ciencias, Universidad de Chile
Centro de Investigación Eutropia

Introducción

Durante Abril de 2015, investigadores del Centro de Investigación Huinay, informaron de una muerte masiva de ballenas en una bahía cercana al Golfo de Penas. Posterior a la denuncia SERNAPESCA organizó con el apoyo de la Armada de Chile y la participación de diversas Instituciones, una visita al área con el fin de evaluar la presencia de intervención antrópica en el suceso, así como colectar información para para investigar con mayor profundidad el evento. Muestras de piel fueron colectadas de la mayoría de los ejemplares. En este reporte se informan los resultados de la investigación de las muestras para clarificar la identificación especie-específico de los ejemplares.

Metodología

Tejido correspondiente a 15 de 39 individuos muestreados fueron enviadas al Laboratorio de Ecología Molecular de la Universidad de Chile.

Extracción de ADN, amplificación, secuenciación y alineamiento de secuencias de región control (D-loop) de ADN mitocondrial

Se realizó la extracción de ADN y posterior amplificación de la región control de ADN mitocondrial (ADNmt) mediante extracción salina. Esta región es comúnmente usada para la identificación de especies de cetáceos por su acelerada tasa de mutación, produciendo así una alta variación entre secuencias y permitiendo discriminar a nivel de especie (Ross *et al.,* 2003). La amplificación de la región control (o D-loop) se realizó utilizando preferencialmente los partidores M13 Dlp1.5 5’-TGTAAAACGACAGCCAGTTCACCCAAAGCTGRARTTCTA-3’ y 8G 5´GGAGTACTATGTCCTGTAACCA (Dalebout *et al.,* 2005) puesto que amplifica un mayor y más informativo tamaño de fragmento. Secundariamente se utilizaron los partidores Dlp-10 5'-CCACAGTACTATGTCCGTATT-3') y Dlp-5 5'-CCATCGWGATGTCTTATTTAAGRGGAA-3' (Baker *et al.,* 1993), que amplifica una región menor. Los productos de PCR fueron purificados y secuenciados en ambos sentidos en Macrogen, Corea del Sur, mediante un

Secuenciador Automático 3730xl. Las secuencias resultantes fueron alineadas y ensambladas manualmente mediante el programa Proseq 2.91 (Filatov, 2002).

Análisis genéticos para identificación de especie

La identificación a nivel de especie se realizó mediante dos programas de análisis comparativo de secuencias (1) "Basic Local Aligment Search Tool(BLAST. www.blast.ncbi.nlm.nih.gov) y (2) DNA Surveillance (Ross *et al.,* 2003). BLAST identifica las regiones de similitud local entre secuencias, comparando nucleótidos o secuencias de proteínas con bases de datos de secuencias y estima el grado de similitud entre ellas. Este programa puede utilizarse para inferir las relaciones funcionales y evolutivas entre secuencias, así como ayudar a identificar a los miembros de las familias de genes. Por su parte, DNA Surveillance es un servicio de determinación de identidad de la especie de cetáceos utilizando métodos filogenéticos. Este programa alinea una secuencia de ADN "problema" contra un set de secuencias de ADN de referencia de especies de cetáceos y luego construye un árbol filogenético (basado en distancias genéticas). El usuario puede entonces hacer inferencias acerca de la identidad de la especie desconocida por su posición en el árbol filogenético (Ross *et al,* 2003). En este último programa, toda la base de datos de la región control mitocondrial presenta al menos una secuencia validada, es decir, proveniente de individuos con identificación corroborada, lo cual proporciona una ventaja comparativa frente a BLAST.

Resultados

De la mayoría de las muestras de tejido recibidas (14 de 15 individuos) se obtuvo suficiente cantidad y calidad de ADN que permitió la amplificación de un fragmento de la región control de ADNmt de mayor tamaño (partidores M13 Dlp1.5 5' y 8G Dalebout *et al.,* 2005). Para el individuo restante se logró amplificar un fragmento de menor número de pares de bases descrito previamente (partidores Dlp-10 y Dlp-5, descrito Baker *et al.,* 1993).

Mediante el programa BLAST se obtuvo un 99% de confiabilidad que todas las secuencias analizadas y comparadas con esa base de datos correspondieron a ballena sei, *Balaenoptera borealis* (Figura 1)**.**

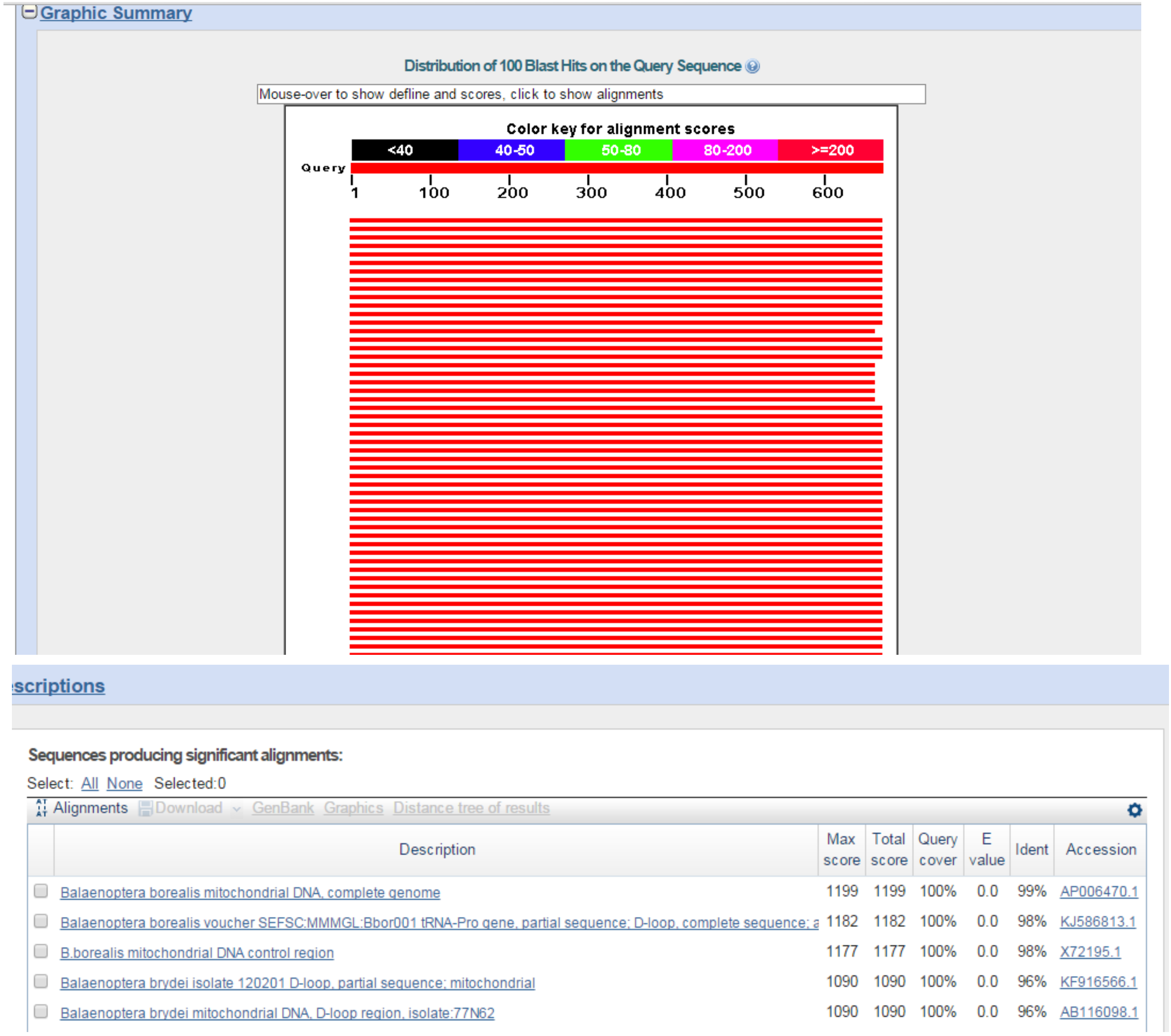

	Description	Max score	Total score	Query cover	E value	Ident	Accession
	Balaenoptera borealis mitochondrial DNA, complete genome	1199	1199	100%	0.0	99%	AP006470.1
	Balaenoptera borealis voucher SEFSC:MMMGL:Bbor001 tRNA-Pro gene, partial sequence; D-loop, complete sequence; a	1182	1182	100%	0.0	98%	KJ586813.1
	B.borealis mitochondrial DNA control region	1177	1177	100%	0.0	98%	X72195.1
	Balaenoptera brydei isolate 120201 D-loop, partial sequence; mitochondrial	1090	1090	100%	0.0	96%	KF916566.1
	Balaenoptera brydei mitochondrial DNA, D-loop region, isolate:77N62	1090	1090	100%	0.0	96%	AB116098.1

Figura 1. Perfil ejemplo de resultado para una de las secuencias analizadas y comparada con la base de datos BLAST. La columna "Ident" proporciona el valor de probabilidad (99%) que la especie corresponda a la especie *Balaenoptera borealis.*

De la misma manera, mediante el programa DNA-Surveillance se obtuvo que la menor distancia genética (5%, Tabla 2), y por ende mayor semejanza, entre las secuencias analizadas y las secuencias de la base de datos correspondientes a *Balaenoptera borealis*. El resultado fue similar cuando se compararon las secuencias tanto a nivel de Orden (Cetácea) (Figura 2) como a nivel de Suborden (Misticeti) (Figura 3).

Tabla 2. Distancias genéticas obtenidas en la comparación de una de las secuencia analizadas (seleccionada al azar como ejemplo) y la base de datos de DNA Surveillance. Se aprecia que la secuencia comparada tiene la menor distancia genética (0,05) (es decir, la mayor semejanza) con la especie *Balaenoptera borealis.*

ID	Common Name	Distance
BborNA5.UA	Sei Whale	0.0501
BedeSA6.UA	Bryde's Whale (Common form)	0.0929
BedeJP1.HY	Bryde's Whale (Kochi form)	0.1066
BmusIC4.UA	Blue Whale	0.1631
BphyIC5.UA	Fin Whale	0.1696
ErobNEP397	null	0.1727
ErobWS03	Gray Whale	0.1729
MnovSEA9	Humpback Whale	0.1756
BacuNPSWFC	North Pacific Minke Whale	0.1771
BacuNA6.UA	North Atlantic Minke Whale	0.1794
BbonSO8.RH	Antartic Minke Whale	0.1855
CmarNZ1.SB	Pygmy Right Whale	0.1887
EglaNA9.SM	North Atlantic Right Whale	0.2126
CheBP03D	Hector's Dolphin	0.2333
EausSP16NP	Southern Right Whale	0.2384
BmysNEPSB1	Bowhead	0.2417
CcomL2.FP	Commerson's Dolphin	0.2443
LobsPE2.FC	Dusky Dolphin	0.2552
LacuNF6.FC	Atlantic White-Sided Dolphin	0.2554
KbreNZ01SP	Pygmy Sperm Whale	0.2573
TtruT2.JW	Bottlenose Dolphin (truncatus form)	0.2711
DcapCA1.PR	Common Dolphin (Long-Beaked)	0.272
DdelCA6.PR	Common Dolphin (Short-Beaked)	0.2788
GgriNZ1.MD	Risso's Dolphin	0.2788
LobINP8.FC	Pacific White-Sided Dolphin	0.283
BbaSW4965	Baird's Beaked Whale	0.2836
GmelNA8.LS	Long-Finned Pilot Whale	0.2949
PspiPE4.PR	Burmeister's Porpoise	0.295

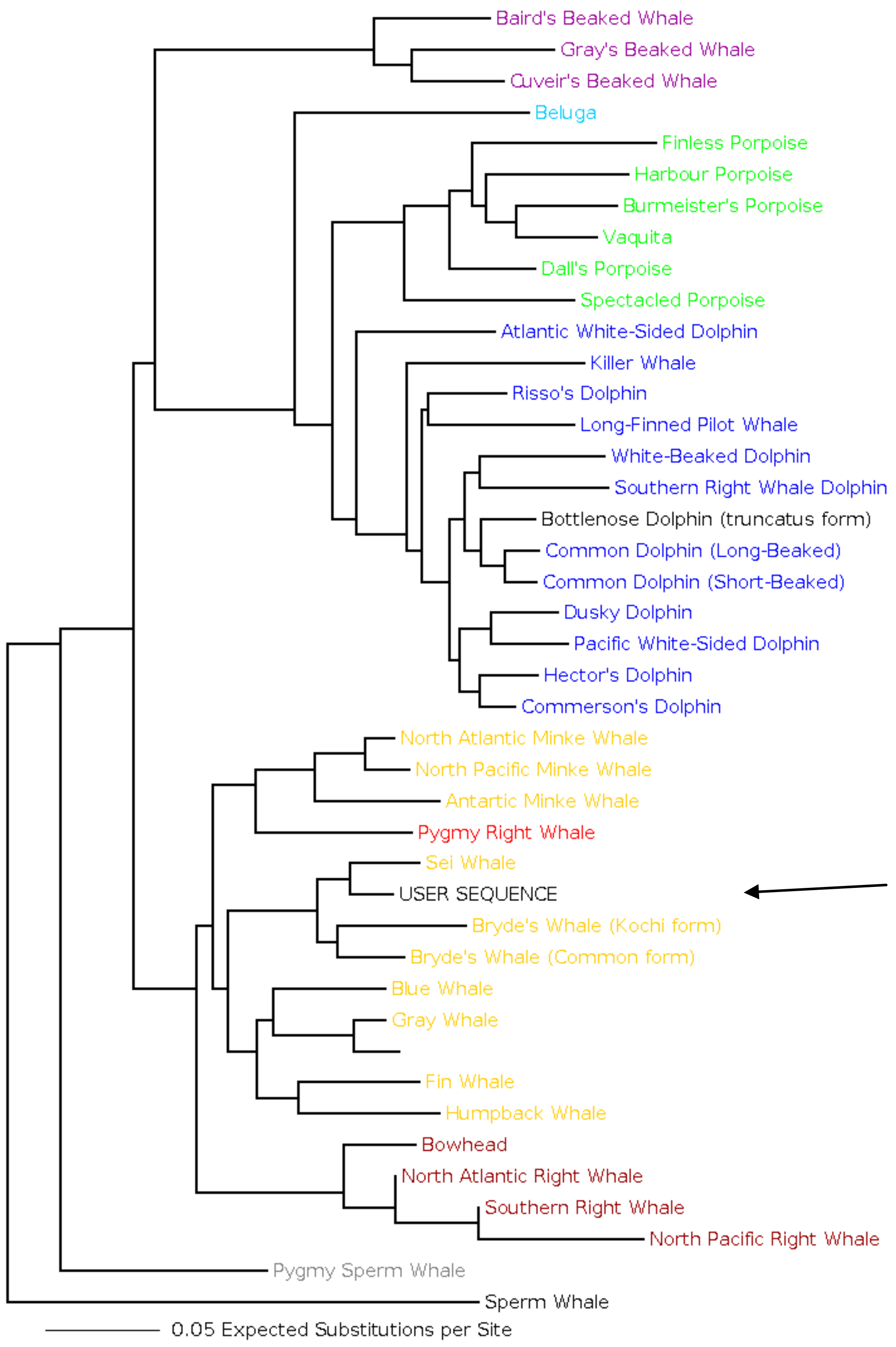

Figura 2. Ubicación de una de las secuencias analizadas (ver flecha) en un árbol de distancia genética construido con información proveniente del orden Cetacea en DNA Surveillance.

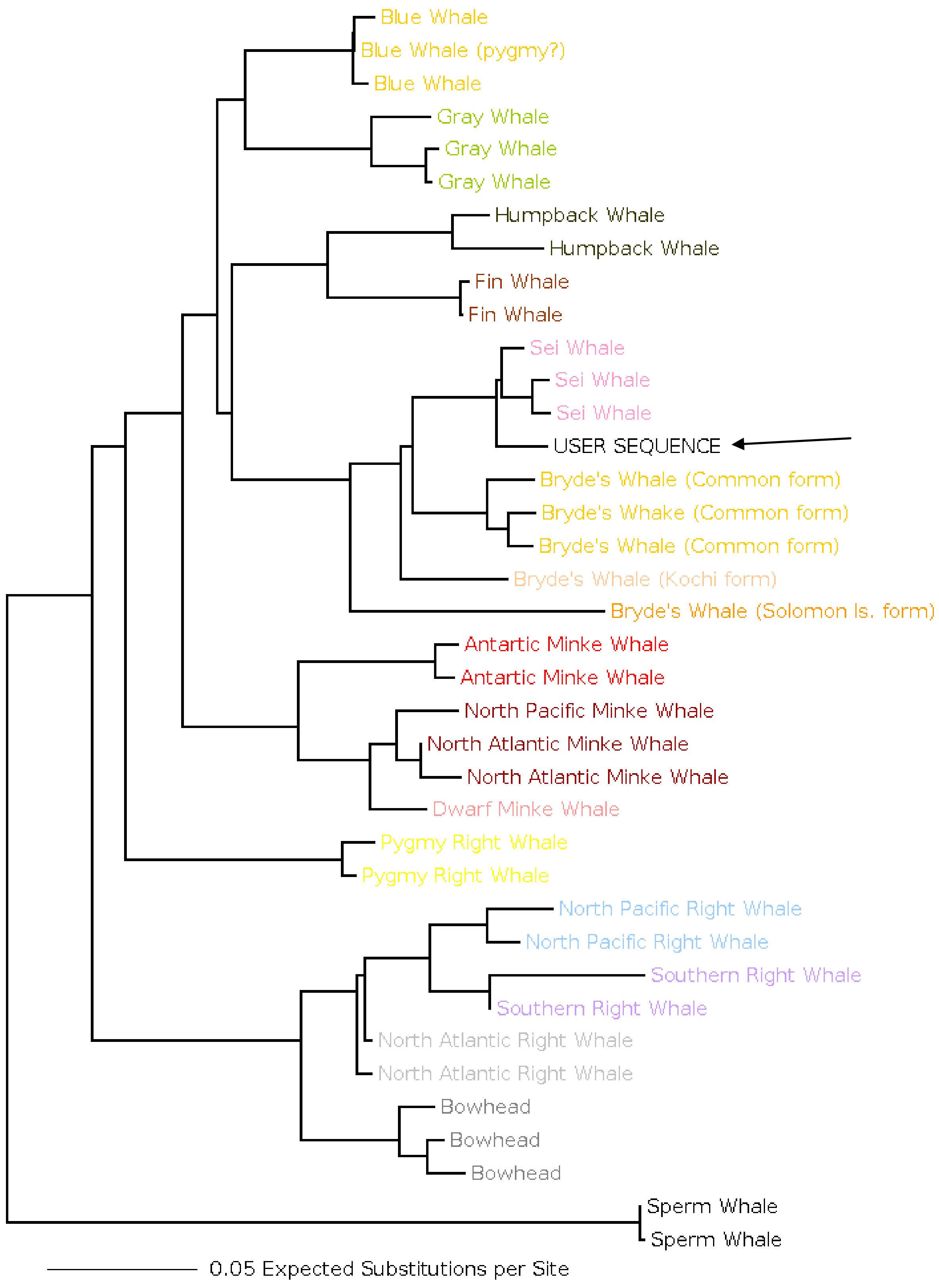

Figura 3. Ubicación de una de las secuencias analizada (ver flecha) en un árbol de distancia genética construido con información proveniente del orden suborden Misticeti en DNA Surveillance.

Literatura Citada

Baker CS, A Perry, L Bannister, M Weinrich, R Abernethym J Calamokidis, J Lien, R Lambersten, J Urban, O Vásquez, P Clapham, A Alling, Aj O Brien and Palumbi. 1993. Abundant mitochondrial DNA variation and world-wide population structure in humpback whales. Proc. Natl. Acad. Sci. U.S.A., 90, 8239–8243.

Dalebout M, K Robertson, A Frantzis, D Engelhaupt, A Mignucci- Giannoni, R Rosario-Delestre, CS Baker. 2005. Worldwide structure of mtDNA diversity among Cuvier's beaked whales (Ziphius cavirostris): implications for threatened populations. Mol. Ecol., 14, 3353–3371.

Filatov, D. 2002. ProSeq: A software for preparation and evolutionary analysis of DNA sequence data sets. Mol. Ecol. Not., 2, 621–624.

Ross, HA, GM Lento, ML Dalebout, M Goode, G Ewing, P McLaren, AG Rodrigo, S Lavery, and CS Baker. 2003. DNA Surveillance: Web-based molecular identification of whales, dolphins and porpoises. Journal of Heredity, 94(2), 111–114.

Referencias

1. Aguayo-Lobo A, Torres D, Acevedo J. 1998. Los mamíferos marinos de Chile: I. Cetacea. Serie Científica INACH 48: 19–159.

2. Aguayo Lobo A. 1974. Baleen whales of continental Chile. Pp: 209 – 217. En: William E. Schevill, Editor. The Whale Problem: A Status Report. Harvard University Press, 419 pp.

3. Aguayo A., J. Acevedo y R. Vargas. 2006. Diversidad de mamíferos marinos en las aguas del Archipiélago de los Chonos (43°39'S – 45°50'S), XI región de Chile. Cienci. Tecnol. Mar, 29 (2): 129 – 145.

4. Baker CS, A Perry, L Bannister, M Weinrich, R Abernethym J Calamokidis, J Lien, R Lambersten, J Urban, O Vásquez, P Clapham, A Alling, Aj O Brien and Palumbi. 1993. Abundant mitochondrial DNA variation and world-wide population structure in humpback whales. Proc. Natl. Acad. Sci. U.S.A. 90, 8239–8243.

5. Dalebout M, K Robertson, A Frantzis, D Engelhaupt, A Mignucci- Giannoni, R Rosario-Delestre, CS Baker. 2005. Worldwide structure of mtDNA diversity among Cuvier's beaked whales (Ziphius cavirostris): implications for threatened populations. Mol. Ecol. 14, 3353–3371.

6. Donovan, G. P. 1991. A review of IWC stock boundaries. Rept. Int. Whal. Commn., Special Issue 13: 39-68.

7. Doucette, G.J., A.D. Cembella, J.L. Martin, J. Michaud, T.V.N. Cole & R.M. Rolland. 2006. Paralytic shellfish poisoning (PSP) toxins in North Atlantic right whales *Eubalaena glacialis* and their zooplankton prey in the Bay of Fundy, Canada. Mar. Ecol. Prog. Ser. 306: 303-313.

8. Durbin, E., G. Teegarden, R. Campbell, A. Cembella, M.F. Baumgartner & B.R. Mate. 2002. North Atlantic right whales, *Eubalaena glacialis*, exposed to paralytic shellfish poisoning (PSP) toxins via a zooplankton vector, *Calanus finmarchicus*. Harmful algae 1: 243-251.

9. Filatov, D. 2002. ProSeq: A software for preparation and evolutionary analysis of DNA sequence data sets. Mol. Ecol. Not. 2, 621–624.

10. Gambel, R. 1985. Sei whale, *Balaenopera borealis*, Lesson 1828. Pp: 155 – 170. En: Sam H. Ridgway y Richard Harrison Editors. Handbook of Marine Mammals. Vol 3. The Sirenian and Baleen whales. Academic Press, London, 369 pp.

11. Geraci, J.R., D.M. Anderson, R.J. Timperi, D.J. St Aubin, G.A. Early, J.H. Prescott & C.A. Mayo 1989. Humpback whales (*Megaptera novaeangliae*) fatally poisoned by dinoflagellate toxin. Can. J. Fish. Aquat. Sci. 46: 1895-1898.

12. Horwood, J. 1987. The sei whale: population biology, ecology & management. Croom Hel, Beckenham. 373 pp.

13. IWC (International Whaling Commission). 1996. Report of the subcommittee on Southern Hemisphere baleen whales. Report of the International Whaling Commission 46: 117-138.

14. Kanda N, Goto M, Pastene LA (2006) Genetic characteristics of western North Pacific sei whales, *Balaenoptera borealis*, as revealed by microsatellites. Mar. Biotechnol. 8: 86–93. DOI: 10.1007/s10126–005–5130–1.

15. Ley 20293 del 25-10-2008; Protege a los Cetáceos e introduce modificaciones a la Ley Nº 18.892 General de Pesca y Acuicultura.Párrafo 5°; DE LA PROTECCIÓN, RESCATE, REHABILITACIÓN, REINSERCIÓN, OBSERVACIÓN Y MONITOREO DE MAMÍFEROS , REPTILES Y AVES HIDROBIOLÓGICAS.

16. Pastene LA, Schimada H. 1999. Report of a sighting survey in Chile's exclusive economic zone with comments on Sei whale distribution. Anales Instituto Patagonia. Serie Cs. Nat. Chile. (2): 51 – 62.

17. Ross, HA, GM Lento, ML Dalebout, M Goode, G Ewing, P McLaren, AG Rodrigo, S Lavery, and CS Baker. 2003. DNA Surveillance: Web-based molecular identification of whales, dolphins and porpoises. Journal of Heredity 94(2), 111–114.

18. Sernapesca, 2014, Fichas resumidas de varamiento de fauna marina de la Unidad de Rescate y Conservación de Especies Acuáticas Protegida del Servicio Nacional de Pesca y Acuicultura.
